# Prodrugs for colon-restricted delivery: Design, synthesis, and *in vivo* evaluation of colony stimulating factor 1 receptor (CSF1R) inhibitors

**DOI:** 10.1371/journal.pone.0203567

**Published:** 2018-09-07

**Authors:** Dawn M. George, Raymond J. Huntley, Kevin Cusack, David B. Duignan, Michael Hoemann, Jacqueline Loud, Regina Mario, Terry Melim, Kelly Mullen, Gagandeep Somal, Lu Wang, Jeremy J. Edmunds

**Affiliations:** 1 Immunology Discovery, AbbVie Bioresearch Center, Worcester, Massachusetts, United States of America; 2 Department of Chemistry, Immunology Discovery, AbbVie Bioresearch Center, Worcester, Massachusetts, United States of America; 3 Department of Drug Metabolism, Pharmacokinetics & Bioanalysis, AbbVie Bioresearch Center, Worcester, Massachusetts, United States of America; 4 Department of Pharmacology, Immunology Discovery, AbbVie Bioresearch Center, Worcester, Massachusetts, United States of America; Universidade do Porto, Faculdade de Farmácia, PORTUGAL

## Abstract

The ability to restrict low molecular weight compounds to the gastrointestinal (GI) tract may enable an enhanced therapeutic index for molecular targets known to be associated with systemic toxicity. Using a triazolopyrazine CSF1R inhibitor scaffold, a broad range of prodrugs were synthesized and evaluated for enhanced delivery to the colon in mice. Subsequently, the preferred cyclodextrin prodrug moiety was appended to a number of CSF1R inhibitory active parent molecules, enabling GI-restricted delivery. Evaluation of a cyclodextrin prodrug in a dextran sodium sulfate (DSS)-induced mouse colitis model resulted in enhanced GI tissue levels of active parent. At a dose where no significant depletion of systemic monocytes were detected, the degree of pharmacodynamic effect–measured as reduction in macrophages in the colon–was inferior to that observed with a systemically available positive control. This suggests that a suitable therapeutic index cannot be achieved with CSF1R inhibition by using GI-restricted delivery in mice. However, these efforts provide a comprehensive frame-work in which to pursue additional gut-restricted delivery strategies for future GI targets.

## Introduction

**Fig 1:**
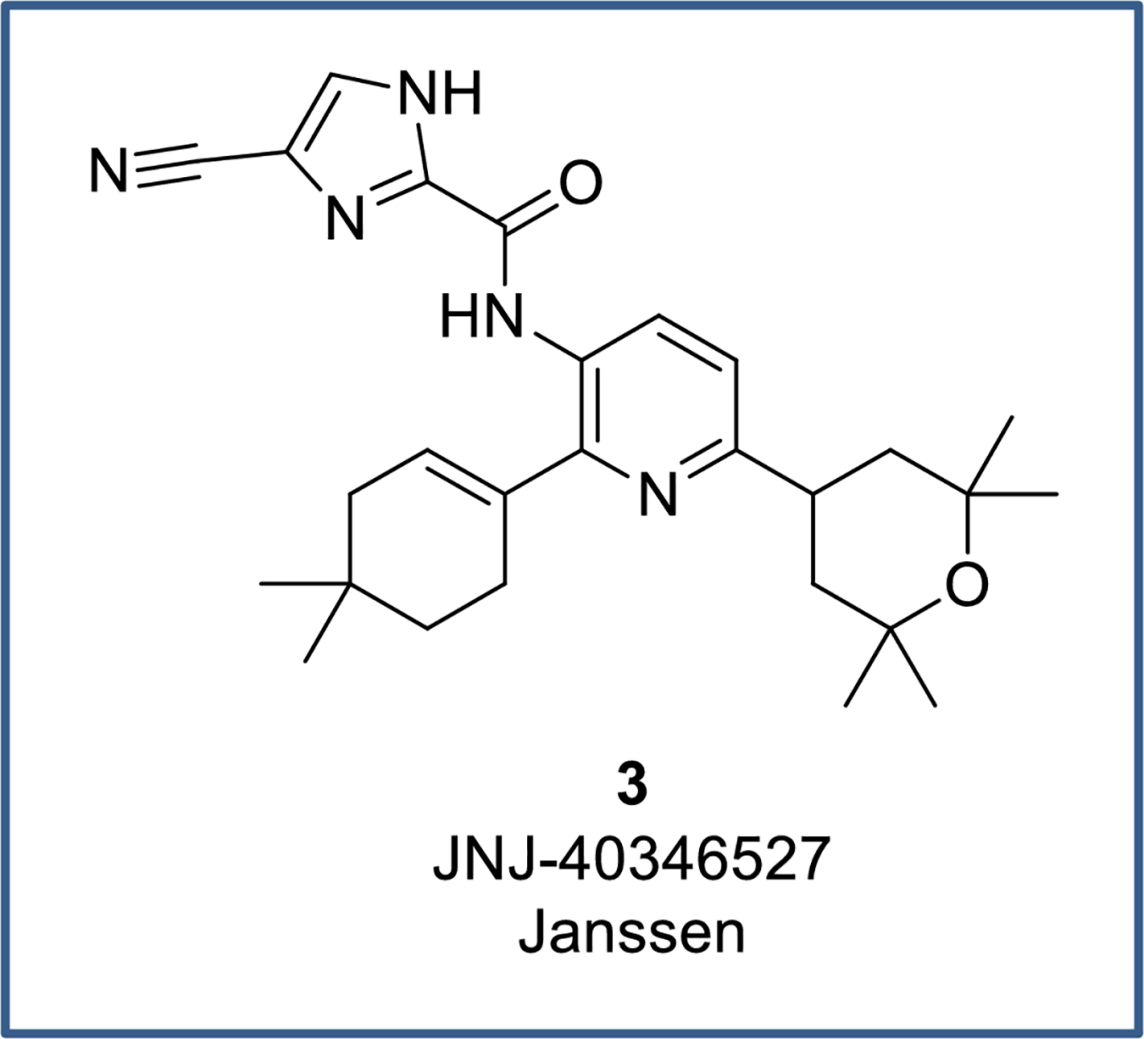
Structure of CSF1R inhibitor from Janssen.

Colony-stimulating factor 1 receptor (CSF1R), a tyrosine kinase that is highly expressed in myeloid cells, including monocytes, macrophages and osteoclasts, regulates their differentiation, proliferation, and survival. CSF1R serves as the receptor for two ligands: colony stimulating factor 1 (CSF1) and interleukin 34 (IL-34). Inhibition of CSF1R to deplete macrophages, either via small molecule intervention or by using antibodies to the receptor or ligands, has been targeted as a potential therapeutic in the field of oncology.[1] Oncologic agents have demonstrated efficacy in xenograft tumor models[2–5] and are advancing to clinical development.[2, 6] Interest in CSF1R for immunological indications has grown[7] as activation of macrophages is believed to play a role in diseases such as rheumatoid arthritis (RA), lupus, and inflammatory bowel disease (IBD).[8–12] Notably, recent clinical data has cast doubts over whether blocking the CSF1R pathway will be efficacious in arthritis. The CSF1R inhibitor JNJ-40346527 (**3**, Fig 1) completed a Phase II trial in disease modifying anti-rheumatic drug (DMARD)-refractory active RA but demonstrated a lack of efficacy in spite of sufficient exposure and systemic target engagement.[13] An anti-CSF1R antibody, FPA-008, is advancing to Phase II clinical trials for RA, and results of this study will help refute or support the role of CSF1R in RA disease.

**Fig 2:**
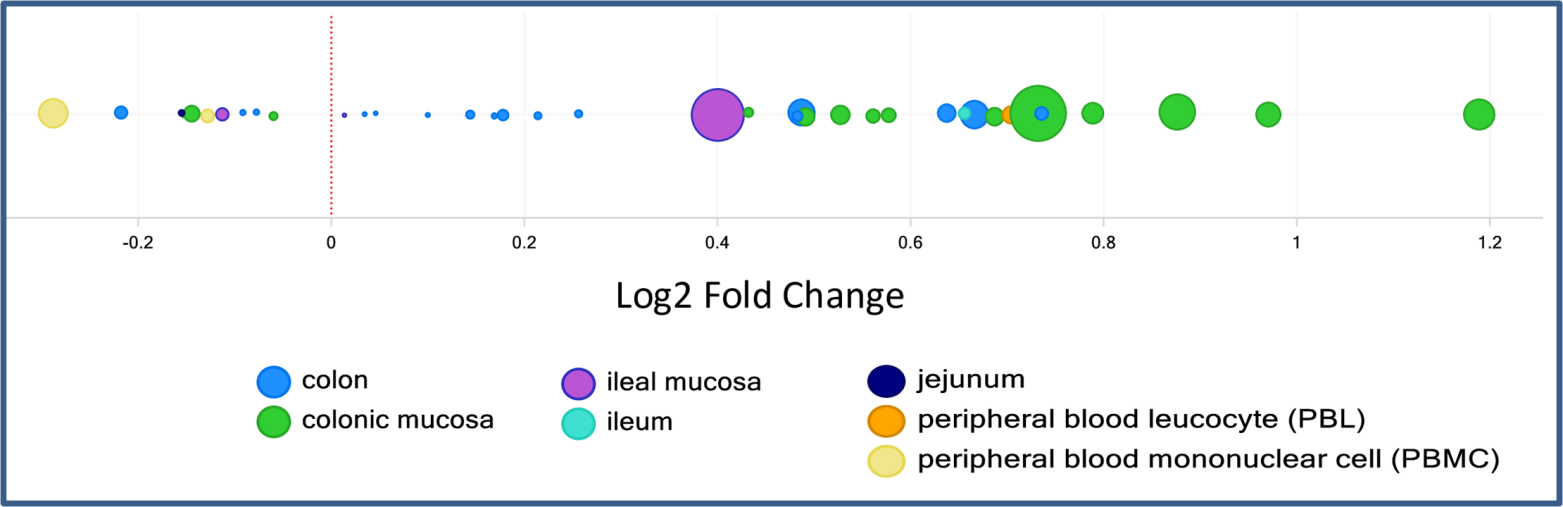
Upregulation of CSF1R gene expression in IBD patients. Data is sized by P-value. The data was extracted from Omicsoft® ImmunoLand® 2015 Q4 release [19].

Analyses of diseased tissue from Crohn’s and ulcerative colitis patients have shown an accumulation of pro-inflammatory macrophages, indicating a potentially pathologic role for this cell type in IBD.[14–16] Human expression data supports a specific role for CSF1R in IBD as CSF1R gene expression is significantly upregulated in the colon and colonic and ileal mucosa of IBD patients (Fig 2).[17] Furthermore, preclinical data showing reduction in clinical and histopathological disease readouts with **3** in a model of murine colitis provides additional evidence supporting the utility of CSF1R inhibition for GI disease.[18]

Despite possible beneficial outcomes, systemic inhibition of CSF1R has the potential to cause undesirable on-mechanism effects that would prevent a sufficient safety index for Crohn’s Disease (CD). One anticipated on-target effect is the depletion of Kupffer cells (liver macrophages). Kupffer cells are responsible for clearance of several short-lived serum enzymes that are typically elevated as a result of liver or skeletal muscle injury. Significant increases in alanine aminotransferase (ALT), aspartate aminotransferase (AST), and/or creatine kinase (CK) have been observed pre-clinically and clinically with anti-CSF1R and anti-CSF antibodies[20–22] and small molecule CSF1R inhibitors.[23, 24] Typically, monitoring of these enzymes is used in clinical practice to indicate liver damage, and this on-target effect of increased serum enzymes would make clinical development challenging for chronically ill IBD patients who may taking other concurrent medication.[25] Other on-target effects are predicted from CSF1R deficient mice, and include reduced fertility, abnormal bone metabolism, and impact on brain development. [26–28] In humans, various mutations in the CSF1R protein kinase domain give rise to a predisposition to hereditary diffuse leukoencephalopathy with neuroaxonal spheroids.[29]

In the context of inflammatory GI disease, systemic CSF1R inhibition would be expected to deplete circulating monocytes and colonic macrophages, both involved in colitis disease process To address the aforementioned on-mechanism safety concerns, we hypothesized that minimizing systemic exposure to a CSF1R inhibitor would improve safety margins by avoiding exposure to the liver, bone, reproductive tract, and brain. Colon-localized delivery of a CSF1R inhibitor, defined as maintaining exposures over the CSF1R cellular EC_50_ in the colon and remaining considerably below in the blood, should not deplete circulating monocytes but may prevent monocytes that traffic into the colon from differentiating into macrophages, and decrease survival and activation of existing tissue macrophages. Our goal was to explore whether sufficient efficacy would be maintained with colon-localized CSF1R inhibition by comparing the effect of local delivery of small molecule CSF1R inhibitors to that of a known systemic CSF1R inhibitor in a preclinical mouse model of colitis.

Two broad strategies for GI-restriction were evaluated: (1) limit oral absorption in the upper GI and (2) maximize hepatic and/or non-hepatic metabolism to inactive metabolites. In terms of limiting oral absorption, two tactics were considered: design of compounds with non-traditional drug-like properties–high molecular weight, hydrophilic compounds,[30] and utilization of prodrugs that are metabolized to release active drug in the colon. For the high metabolism approach, strategies exist to maximize metabolism to inactive metabolites including engagement of intestinal or blood esterases and amidases. Consideration was given to identifying compounds that are substrates of cytochrome P450 3A4 (CYP3A4). This drug metabolizing enzyme is highly expressed along the GI tract in human and rodents.[31] Thus, compounds known to undergo metabolism by CYP3A4 could be metabolized to inactive metabolites upon absorption into intestinal epithelial cells, resulting in minimized systemic exposure of active parent compound. However, a key concern around this approach was the potential for saturation of CYP3A4, as well as potential drug-drug interactions associated with co-administered CYP3A4 substrates or inhibitors, and therefore this approach was deprioritized.

The high expression of P-glycoprotein (P-gp or Multi-drug Resistance 1 (MDR1) protein) efflux transporter along the GI tract[32, 33] suggested an additional potential tactic to restrict systemic exposure. Following absorption across mucosal tissue, compounds that are P-gp substrates could be actively transported back into the lumen of the small intestine thus reducing systemic exposure. Although some compounds we studied were P-gp substrates, the effect on absorption was not definitively assessed. In addition, P-gp is also expressed in distal colon which could limit exposure to this target organ. As a result, P-gp inhibition was not selected as a strategy of focus.

Restricted by the limitations of mouse physiology that preclude both the use of capsule-based formulation (due to size) and pH-modulated release (due to lack of an appreciable pH gradient in the mouse GI tract[34]), the prodrug approach to limit oral absorption in the upper GI tract was selected. Several reviews have highlighted the assortment of prodrug options that may be amenable to lower GI delivery, including dimethylglycine, valine, glucoside, glucuronide, cyclodextrin, and dextran adducts.[35–37] Typically, large, polar molecules are poorly absorbed in the small intestine and arrive intact in the colon where active parent may then be liberated by various cleavage mechanisms. In spite of this breadth of historical prodrug work, a comprehensive comparison for different prodrugs conjugated to the same active parent molecule has not been studied. An assessment of the relative effectiveness of each prodrug to selectively deliver active compound to the GI tissue is therefore lacking. An encouraging precedent for colon-specific delivery of a cyclodextrin prodrug is seen with an orally dosed prednisolone-cyclodextrin conjugate that demonstrated efficacy in a rat colitis model.[38] The prodrug gave comparable effects to prednisolone on colonic disease score, and demonstrated a significantly improved thymus:body weight ratio, a measure of side effects induced by systemic exposure.

## Results and discussion

**Fig 3:**
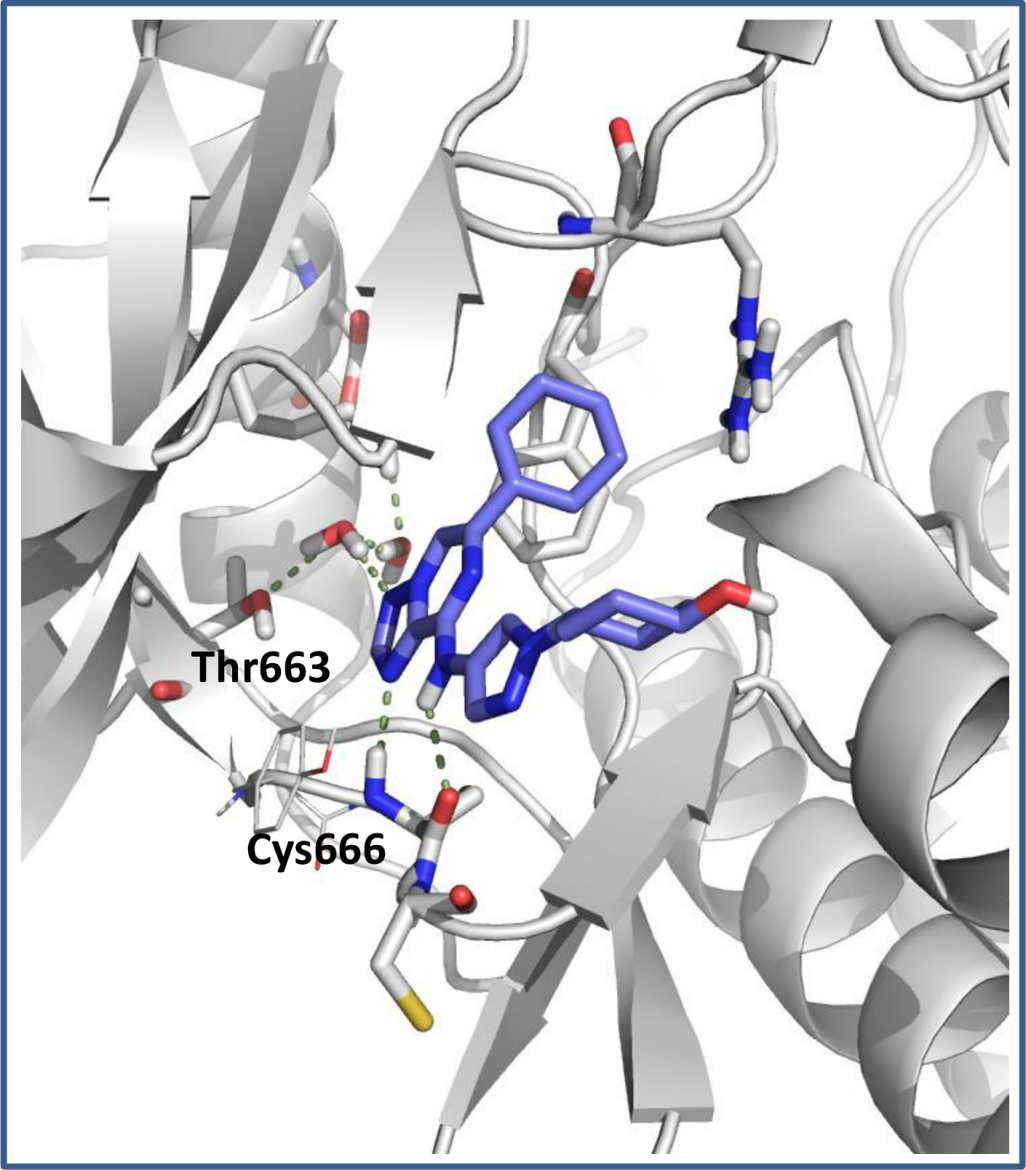
Model of compound 1 with CSF1R. Modeled using the Schrodinger Maestro Glide docking into a grid generated from the CSF1R X-ray crystal structure (PDB Code: 2I0Y) followed by optimization with MM-GBSA [39].

**Fig 4:**
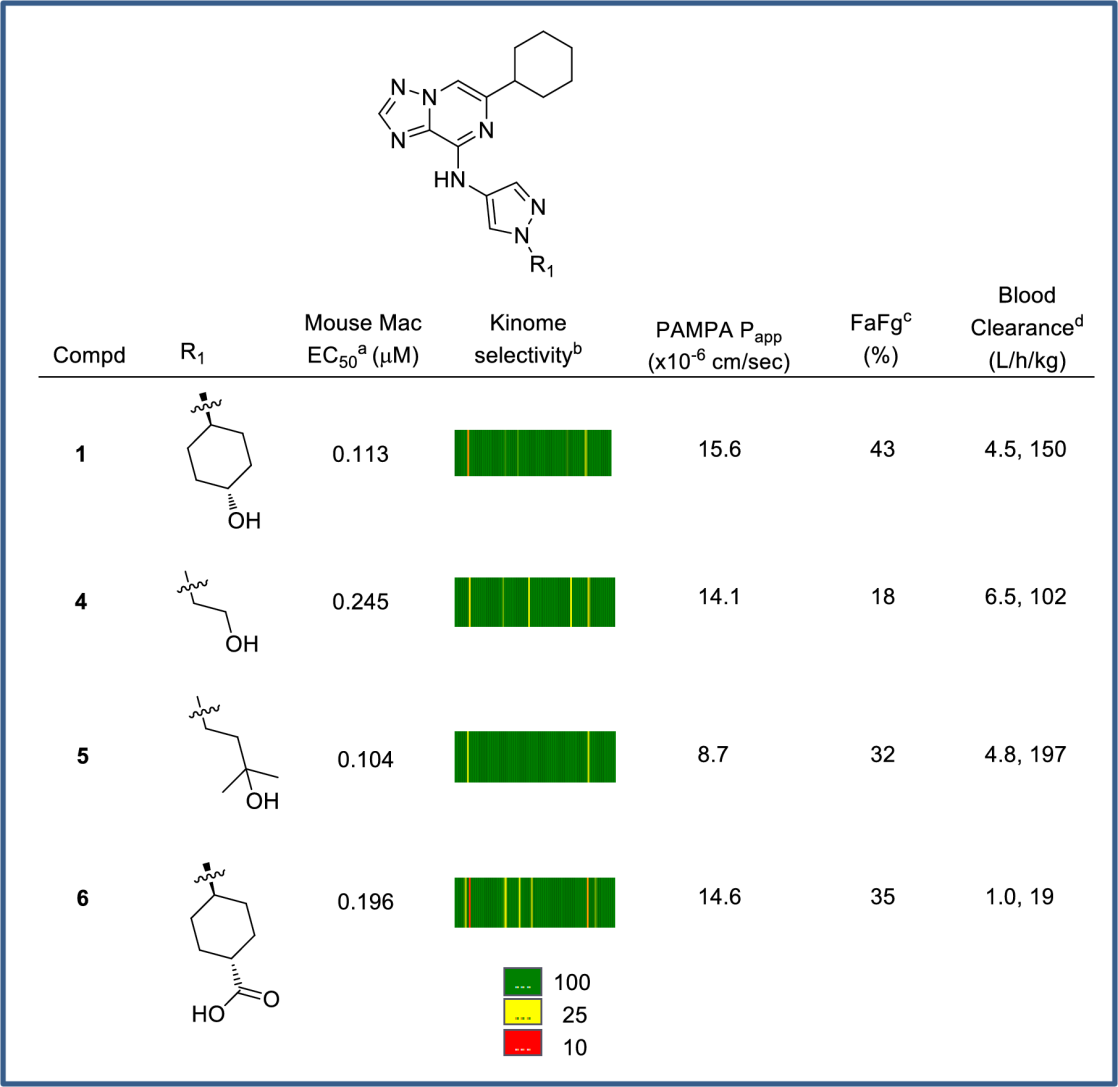
Prodrug candidates. ^a^ Mean from n ≥3 experiments. ^b^ The kinome selectivity data for a panel of 80 kinases are colored by fold selectivity over CSF1R IC_50_, with ≤10-fold selective in red and a color gradient to ≥100-fold selective in green. ^c^ Mean of 1 (compound **1**, **4** and **5**) or 3 (compound **6**) experiments as described in Materials & Methods. ^d^ Mean values determined after a 1 mg/kg IV dose (N = 2–3). Data presented as total clearance and unbound clearance (CL_b_/fu_b_), respectively.

To enable the preparation of prodrug derivatives, we selected four parent compounds (**1**, **4**, **5**, **6**; CSF1R enzyme IC_50_ < 0.010 μM). These CSF1R inhibitors afford a classical two-point hinge binding arrangement with the NH and C = O of Cys666 as a consequence of the exocyclic NH and the adjacent triazole N. In addition, a water-mediated interaction from the remaining non-bridging triazole N to gatekeeper Thr663 is proposed (Fig 3). The pyrazole resides in a water-accessible region that tolerates a variety of substituents, and the cyclohexyl group provides an optimal fit under the Gly-rich loop. Each compound exhibited good CSF1R cellular potency (EC_50_ = 0.104–0.245 μM) in a CSF-induced mouse macrophage differentiation assay, and generally demonstrated at least 10x selectivity when CSF1R enzyme activity was assessed against our internal kinome screening panel (Fig 4; see Methods for kinases tested). The four compounds exhibited high PAMPA permeability, P-gp efflux ratios <2 (suggesting they are not P-gp substrates), and low to moderate absorption (FaFg). This allowed for an assessment of FaFg on penetration into colon tissue and access to the lamina propria where the majority of inflammatory monocytes and macrophages accumulate in diseased GI tissue. The compounds also exhibited low to high systemic clearance.

In order to evaluate colon exposure, the compound concentration in mouse distal colon homogenate was compared to plasma levels, and a maximal ratio of unbound colon:plasma exposure was favored. Although we were most interested in the compound concentration in the lamina propria region of the distal colon, current limitations in tissue isolation methods with mouse colons and methods to quantify in this specific region led to reliance on whole colon homogenate concentrations as a surrogate for concentrations in the lamina propria. To reduce the possibility of fecal contamination impacting measured levels of drug in colon tissue homogenate samples, the isolated colons were flushed thoroughly with PBS prior to snap freezing. The necessity of using whole colon tissue concentrations as a surrogate has been described in the literature. A recent report studying colon tissue concentrations of raltegravir in rats, eloquently described the challenges faced with total tissue measurements, where complete colon tissue drug levels were used as a substitute measurement of gut-associated lymphoid tissue (GALT) concentrations.[40] Moreover, exposure measurements from total tissue homogenate have been used in the antiretroviral field to compare rectal tissue exposure with blood plasma concentrations in human clinical trials.[41–45]

**Fig 5:**
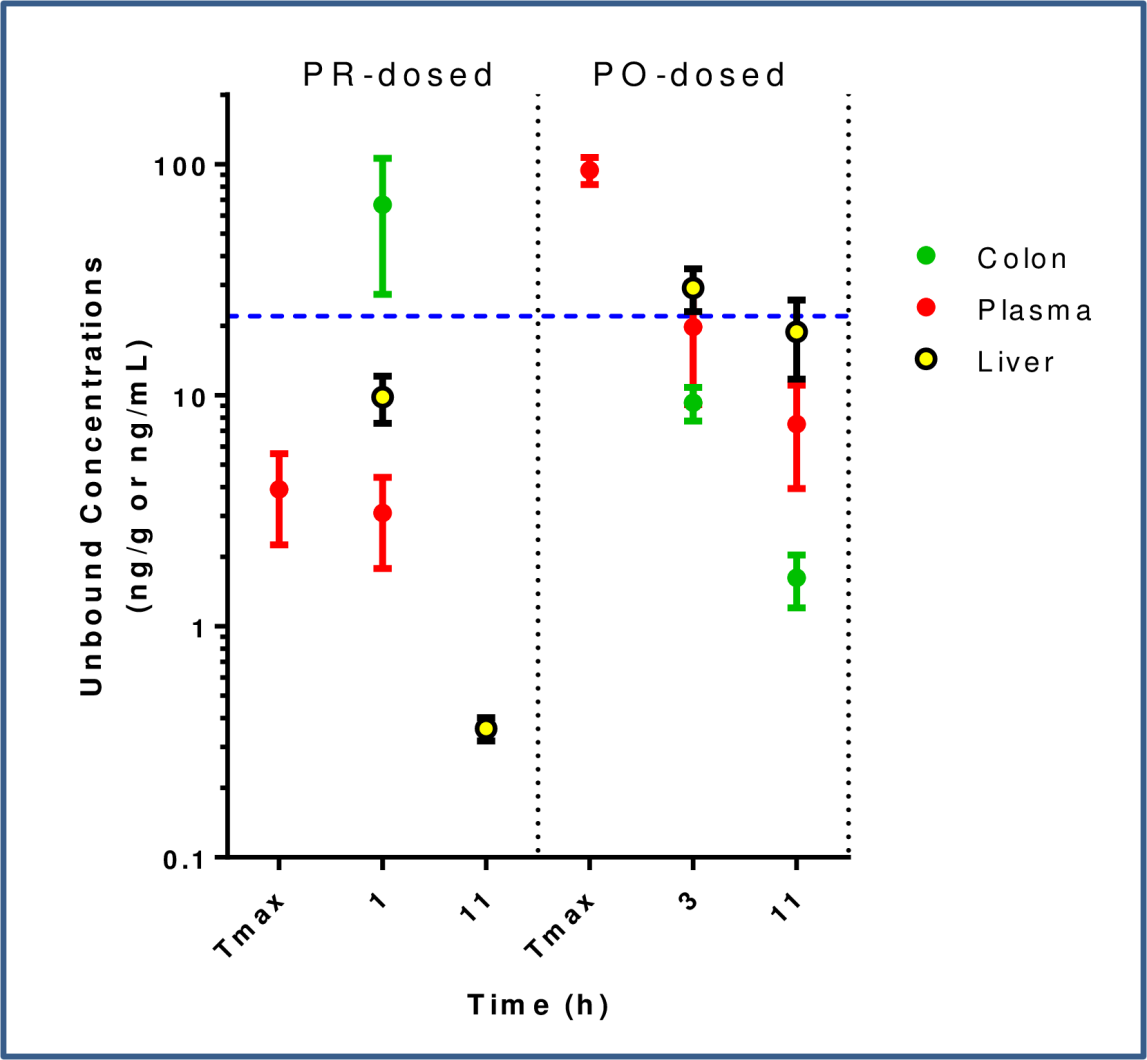
Unbound concentration (ng/mL for plasma; ng/g for colon and liver) of 1 over time after 30 mg/kg PO or PR dosing. The cellular EC_50_ corrected for binding in 10% FBS (22 ng/mL) is represented by the horizontal dotted blue line. Tmax = plasma Tmax.

To simulate the release of active parent molecule in the colon after oral dosing of a prodrug, active parent **1** was initially dosed per rectal (PR) and compared to oral dosing (PR versus PO, 30 mg/kg dose). Compound exposure was measured in colon and liver tissue homogenate, and in plasma (Fig 5) at the estimated colon Cmax (1 h for PR dosing, 3 h for PO dosing) and Clast (11 h). The unbound fraction (fu) was measured in each tissue compartment as this value can vary substantially due to lipid and protein makeup. The fu was used to calculate unbound tissue concentrations for all comparisons. Mean colon unbound Cmax was significantly higher with PR than with PO dosing (67 ng/g and 9 ng/g, respectively) and the 1 h colon exposure exceeded the cellular EC_50_ while the 1 h plasma exposure did not. The colon:plasma and colon:liver ratios at 1 or 3 h were dramatically increased with PR dosing (colon:plasma - 22x versus 0.5x; colon:liver - 7x versus 0.34x). Lack of compound detection in the colon at the terminal time point after PR dosing was attributed to the challenge of maintaining a rectally-dosed solution within the colon in active mice for sufficient time to enable the extended absorption that would be anticipated with oral dosing. The ability to achieve high colon and low systemic exposure with PR dosing confirmed that absorption from the colon is indeed less than from the small intestine[30] and built confidence that prodrug delivery of compound to the colon would give compelling colon levels of drug and low levels in circulation.

**Fig 6:**
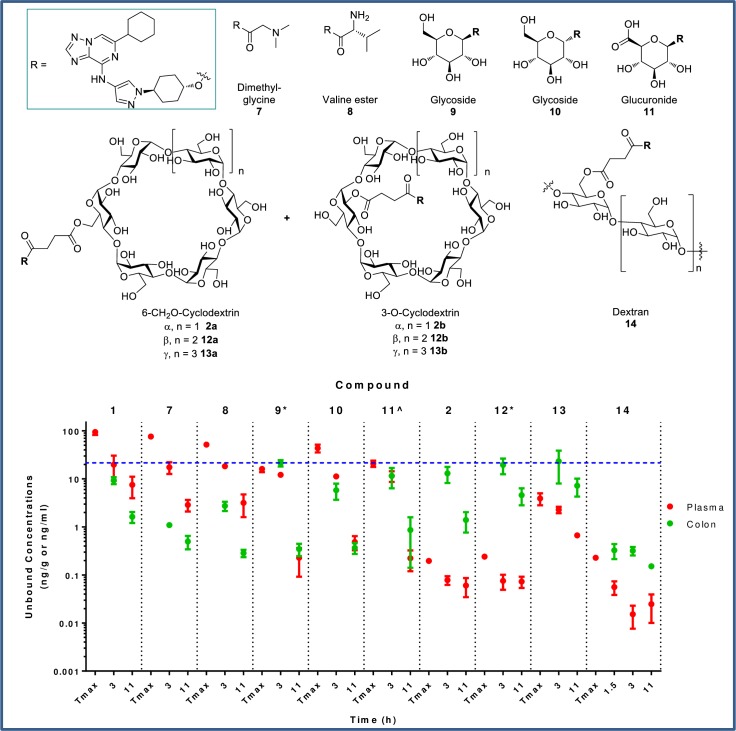
Unbound concentration (ng/mL for plasma; ng/g for colon) of 1 at 3 and 11 h after PO dosing of a 30 mg/kg dose equivalent of prodrugs 2 and 7–14. Compounds **2**, **12**, and **13** were dosed as mixtures of isomers (ratio of **a:b** = 82:18 for **2**; 43:57 for **12**; and 70:30 for **13**). Cmpd **2** is reported as the mean of two separate studies. *Dose = 35 mg/kg, ^Dose = 20 mg/kg. Cellular EC_50_ corrected for binding in 10% FBS (22 ng/mL) is represented by the horizontal blue line. Tmax = plasma Tmax.

Multiple prodrugs can be prepared from a single active compound bearing an appropriate synthetic attachment point. Given the favorable preliminary exposure data with PR dosing, secondary alcohol **1** was selected as the prototype parent molecule from which a broad range of prodrug conjugates (**2**, **7–14**) were prepared (Fig 6). Each prodrug was evaluated for colon and plasma exposure of active parent **1** at 3 and 11 h following oral administration of a dose adjusted to equal a 30 mg/kg dose of **1**. The simple dimethylglycine (**7**) and valine ester (**8**) analogs afforded no advantage over dosing the active parent directly, resulting in significantly higher plasma versus colon drug levels. These “drug-like” derivatives (molecular weight (MW) 466–480; total polar surface area (tPSA) 102–125) are likely absorbed in the small intestine prior to intestinal or plasma esterase-mediated release of active parent. Prodrug **7** is not stable in plasma and no prodrug levels were measurable in plasma after oral dosing. Prodrug **8** was detected, at ~10x lower plasma concentrations than active parent **1**. Oral doses of glucoside and glucuronide analogs **9**–**11** resulted in approximately equivalent amounts of active parent **1** in both colon and plasma. Plasma levels of prodrug **9** were ~100x less than active parent, while no measureable plasma levels of **10** and **11** were observed. The moderate increase in molecular weight (543–557) and polarity (PSA 172–189) may account for reduced absorption of **9**–**11** in the small intestine. For **8**–**11**, relatively low levels of prodrug in feces were measured (3–30% of active parent in feces) suggesting efficient conversion of the prodrug to parent drug. Superior colon:plasma ratios were obtained upon oral administration of succinate-linked cyclodextrin prodrugs **2**, **12**, and **13** (as inseparable mixtures of the 6-*O* and 2-*O* regioisomers) in which unbound colonic levels of **1** at 3 h were at or near the CSF1R cellular EC_50_ and colon:plasma ratios of up to 260-fold and liver:colon ratios of 17-30x were achieved with the α- and β-variants. The large, highly polar nature of these compounds (MW 1436–1760; tPSA 580–738) should preclude absorption of prodrug in the small intestine whereas the low levels of active parent **1** that are detectable in the plasma are ostensibly due to colonic absorption of **1** after microflora-mediated cyclodextrin cleavage. High levels of uncleaved cyclodextrin **2** were detected in the feces (~2-fold higher than levels of active parent in feces). The dextran prodrug (**14**), also attached via a succinate linker, gave low plasma levels of parent drug as expected due to both large size (MW ~70,000) and high polarity (tPSA >> 800), however colonic levels were also low and well below the CSF1R cellular EC_50_. The low colon levels may not be attributed to reduced conversion to active parent as ~20-300x higher levels of active parent **1** in the feces than in the colon tissue homogenate were observed from 3–11 h. Overall, the cyclodextrin prodrugs were preferred for selective delivery of **1** to the colon, and as such were investigated with further analogs.

**Fig 7:**
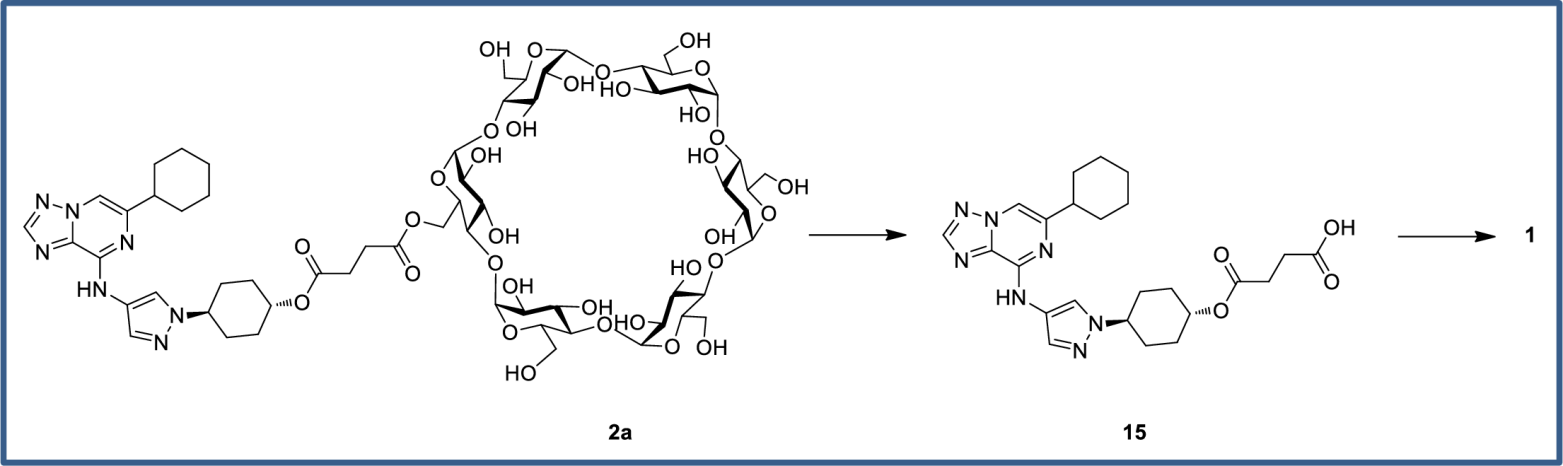
Proposed mechanism of cyclodextrin prodrug cleavage.

The mechanism of cleavage of the cyclodextrin prodrugs is hypothesized to occur via a two-step process (Fig 7). First, bacterial esterases cleave the ester linkage to the cyclodextrin, releasing the hemi-succinate **15**. Although able to inhibit CSF1R enzyme activity, **15** is not stable in mouse plasma *in vitro* and a PK experiment dosing the hemi-succinate **15** IV or PO (1 mg/kg) confirmed the degradation and release of **1** with no detectable plasma levels of **15** at any time point evaluated (from 0.5 to 12 h). Thus it is unlikely that **15** would contribute meaningfully to any *in vivo* efficacy. It is conceivable that the order of degradation steps could be reversed–ester hydrolysis to give **1** directly and subsequent cleavage of succinic acid from the cyclodextrin. Alternatively, hydrolysis of the sugar moiety into smaller saccharide conjugates may occur prior to linker cleavage as was reported from a study on *n*-butyric acid directly linked to β-cyclodextrin.[46]

**Fig 8:**
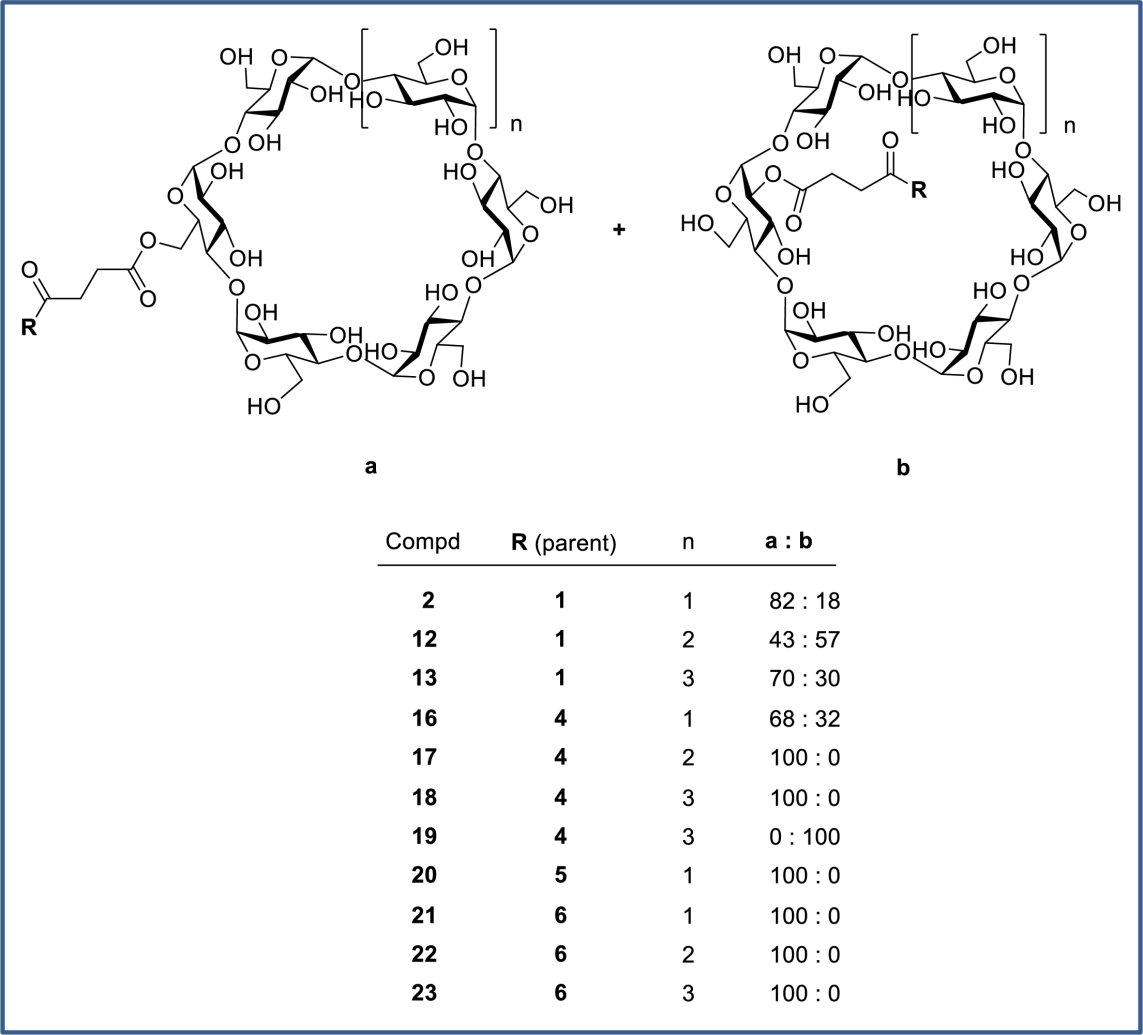
Cyclodextrin prodrugs 2 and 12–23.

**Fig 9:**
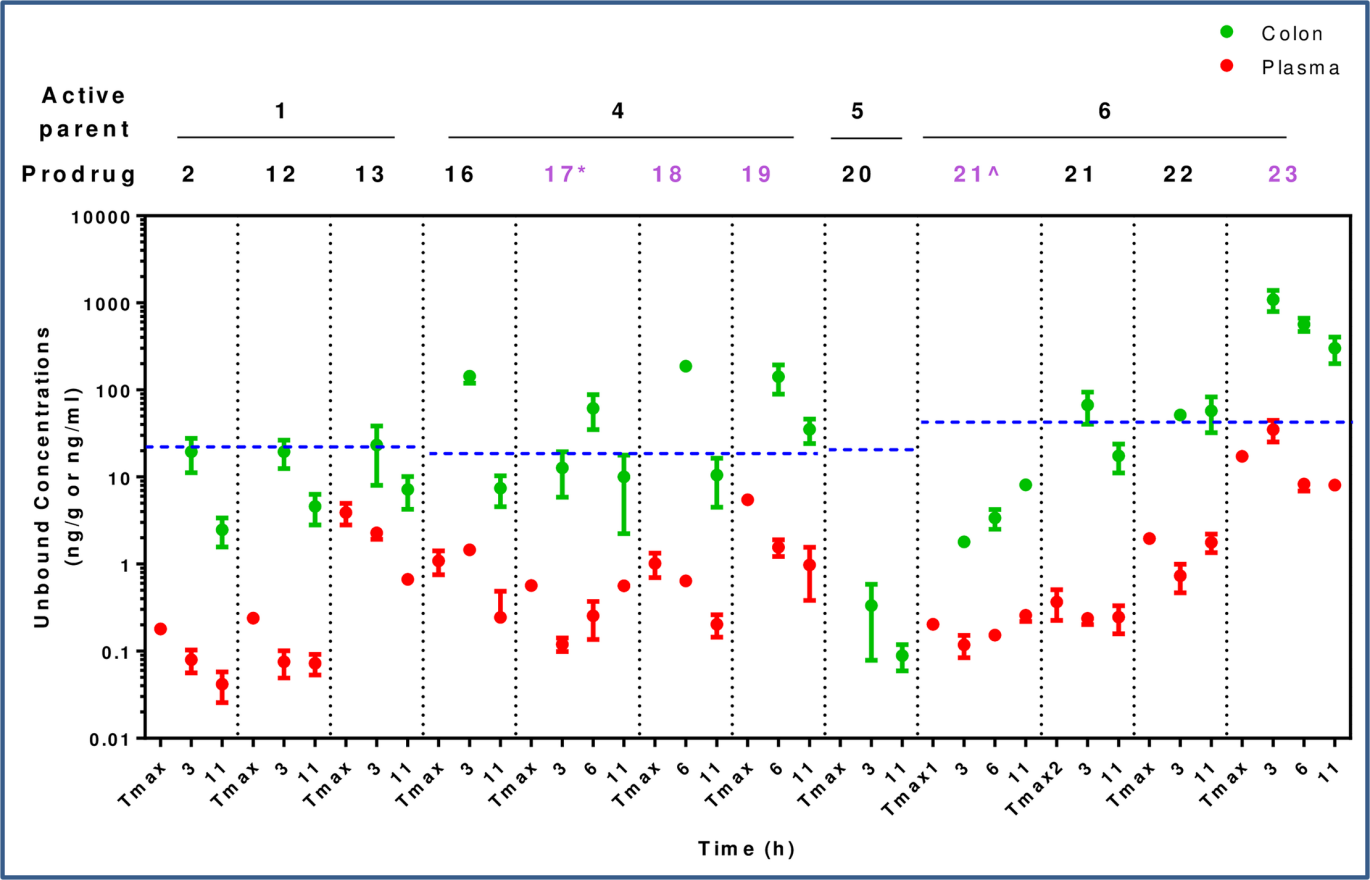
Unbound concentration (ng/mL for plasma; ng/g for colon) of active parent compounds 1 and 4–6 at 3 and 11 h after PO dosing of a 30 mg/kg dose equivalent of cyclodextrin prodrugs 2, 12, 13, and 16–23. Prodrugs were dosed to CD1 male (black) or C57BL/6 female (purple) mice (N = 3). Tmax = plasma Tmax. Cellular EC_50_s for **1**, **4**, **5**, and **6** adjusted for binding in 10% FBS (22, 19, 21, and 42 ng/mL, respectively) are represented by dashed blue lines. *Dose = 25 mg/kg; ^Dose = 32 mg/kg.

Cyclodextrin analogs of the primary alcohol **4**, tertiary alcohol **5**, and carboxylic acid **6** (Fig 8) were prepared and profiled to compare the utility of these alternative prodrug attachment linkers with that of secondary alcohol **1** (Fig 9). In several cases, an inseparable mixture of a- and b-attached cyclodextrins were isolated (**2**, **12**, **13**, **16**) and in other cases only one isomer was observed (**17**, **20**–**23**). In the case of the primary alcohol **4**, both the a- and b-linked cyclodextrins (**18** and **19**) were observed and separable, *vide infra*. The cyclodextrin derivatives **16**–**19** of primary alcohol **4** all gave high colon:plasma exposure with coverage at or above the cellular EC_50_ at 3 or 6 h, and **19** maintained levels of free drug close to the EC_50_ for an extended duration. The relatively high levels of **4** in colon occurred despite its low FaFg (18%). The α-cyclodextrin analog of tertiary alcohol **5** gave minimal colonic exposure, thus additional prodrug derivatives of the tertiary alcohol were not pursued. For prodrugs of the carboxylic acid parent **6**, the γ-cyclodextrin **23** was favored, providing colonic exposure well above the cellular EC_50_ while maintaining a preferred colon:plasma ratio (~30–70 from 3–11 h). The α- and β-cyclodextrin comparators (**21** and **22**, respectively) gave substantially lower overall exposures in the colon. Plasma concentrations of **6** were generally low despite its low systemic clearance and plasma concentrations tended to increase with colon exposure.

**Fig 10:**
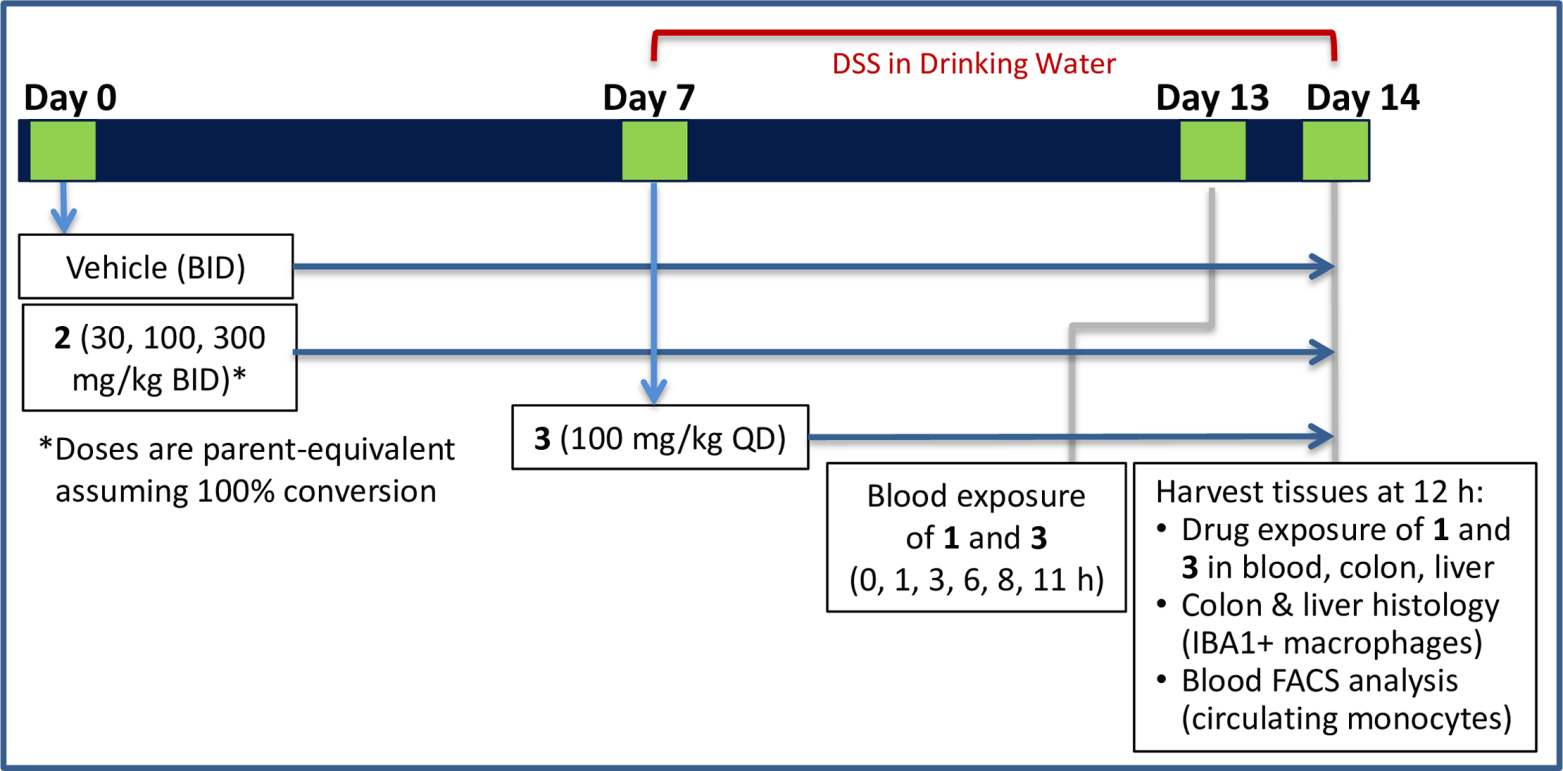
Mouse DSS experimental design.

From the first set of prodrugs prepared with secondary alcohol **1** (**2**, **12**, **13**), α-cyclodextrin **2** was chosen for *in vivo* profiling based on acceptable total colon concentration approaching the total cellular EC_50_, sufficient window of colon:plasma exposure, and relative ease of synthesis to access multi-gram quantities as required to enable *in vivo* studies. A mouse dextran sodium sulfate (DSS)-induced model of colitis was selected due to significant macrophage component and up-regulation of the receptor (CSF1R) and its ligands (CSF1 and IL34) (internal data, not shown). Prodrug **2** was dosed orally twice a day from day 0 to day 14 (30, 100, and 300 mg/kg dose equivalent, Fig 10). The reasoning for providing pro-drug **2** one week prior to initiation of DSS administration was to enable CSF1R-mediated depletion of gut macrophages prior to providing DSS and ensuing inflammatory insult, as the exact contribution resident macrophages have to DSS-mediated disease is unknown. The systemic CSF1R inhibitor **3** was used as a positive control, administered at 100 mg/kg (QD) from day 7–14, as it had historically shown robust efficacy in a mouse DSS model without the need for 1-week pre-treatment dosing regimen. Disease effect on the colon was measured in two ways: a pharmacodynamic readout of the change in macrophage numbers (% of the mucosa that was IBA1+ (ionized calcium-binding adapter molecule 1)[47]) in the colon by image analysis, and a functional readout measuring erosion length in the colon by histology. Systemic effects on liver macrophages were measured by image analysis (% IBA1+ cells) of liver tissue, and on circulating monocytes by fluorescence-activated cell sorting (FACS) to determine depletion of a non-classical monocyte subtype (CD11b+ CD11c+ MHCII-) as well as a classical (CD11b+ CD11c- MHCII-) population. In addition to terminal exposures and PD/efficacy readouts, exposure of **1** in colon, liver, and blood was measured in a separate study after a single dose and after 7 days of BID dosing to evaluate whether drug accumulation occurred prior to DSS administration.

**Fig 11:**
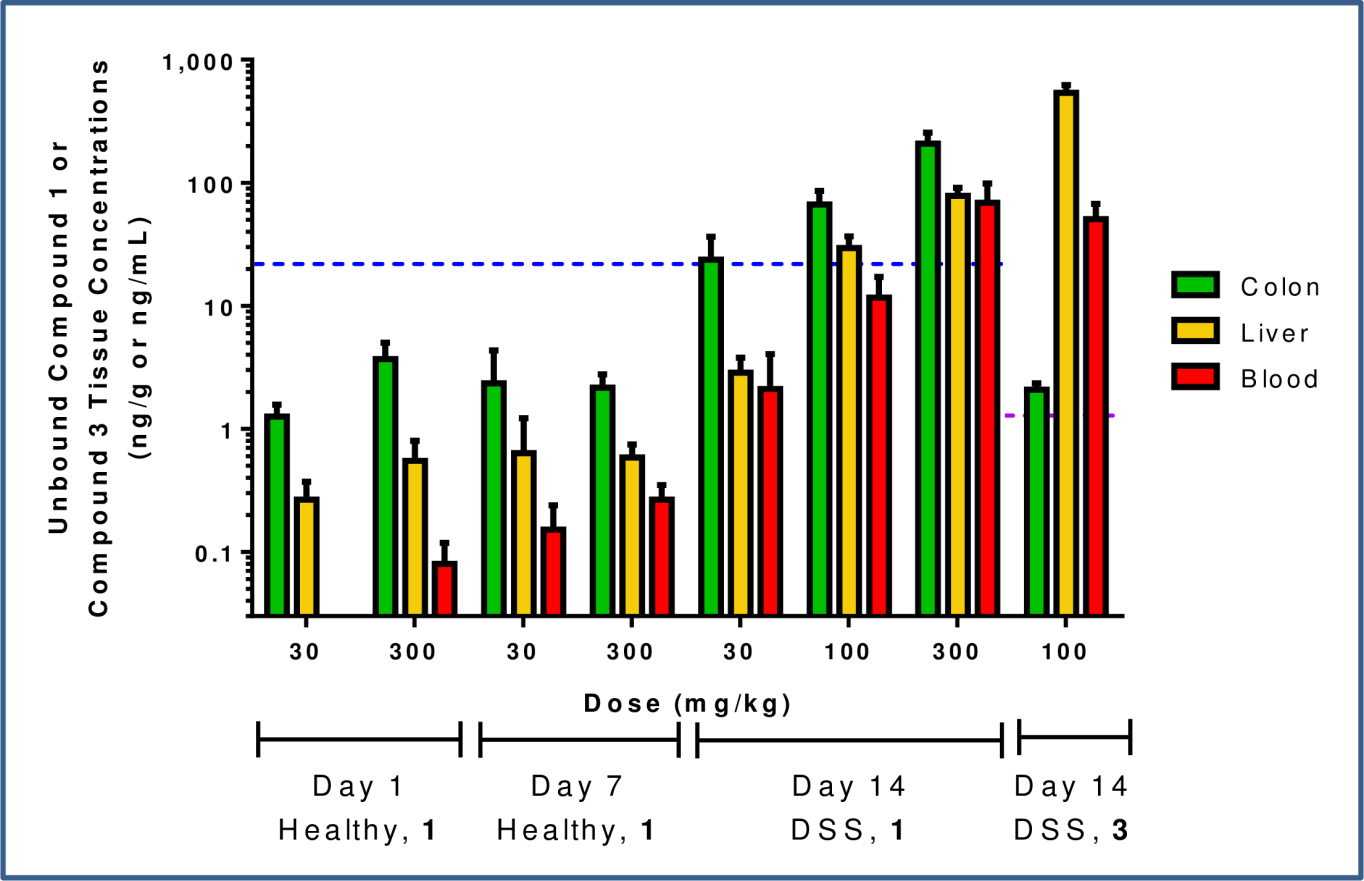
Unbound colon, liver, and blood concentrations of compound 1 in healthy and DSS mice at 11 h (Day 14 DSS) or 12 h (Day 1, Day 7 Healthy) after multi-day dosing of 2. Also shown are unbound concentrations of compound **3** in DSS mice. Cellular EC_50_ corrected for binding in 10% FBS is represented by a dashed blue line (compound **1**) or a purple line (compound **3**).

Compared to exposures following a single dose, after 7 d of BID dosing of **2** in healthy animals no significant accumulation of **1** in the colon or liver was observed, while a modest accumulation was noted in the blood (~3x) in the 300 mg/kg dose group (Fig 11). The data suggests that maximal absorption of active parent is generally achieved at 30 mg/kg in the presence of intact GI epithelium and no benefit is gained with increased dose. Interestingly, in mice subsequently exposed to DSS for 7 days with BID dosing of prodrug **2**, dramatic increases in colon and liver exposures of parent **1** were observed, presumably resulting from loss of epithelial integrity and increased permeability of the GI barrier due to DSS-mediated chemically induced damage. Although blood exposures were higher with DSS treatment than in naïve animals, at 30 mg/kg the mean unbound colon exposure was at cellular EC_50_, while unbound blood exposure was 10x lower than EC_50_ (corresponding to a colon:blood ratio of 11 at this dose) and the 100 mg/kg treated group maintained coverage of cellular EC_50_ in the colon while remaining below in the blood. These results show colon-restricted exposure and therefore allow for the determination of whether GI-restricted exposure of CSF1R inhibitor could enable local depletion of colonic macrophages in the DSS study, while sparing systemic PD effects such as liver macrophage and blood monocyte depletion.

**Fig 12:**
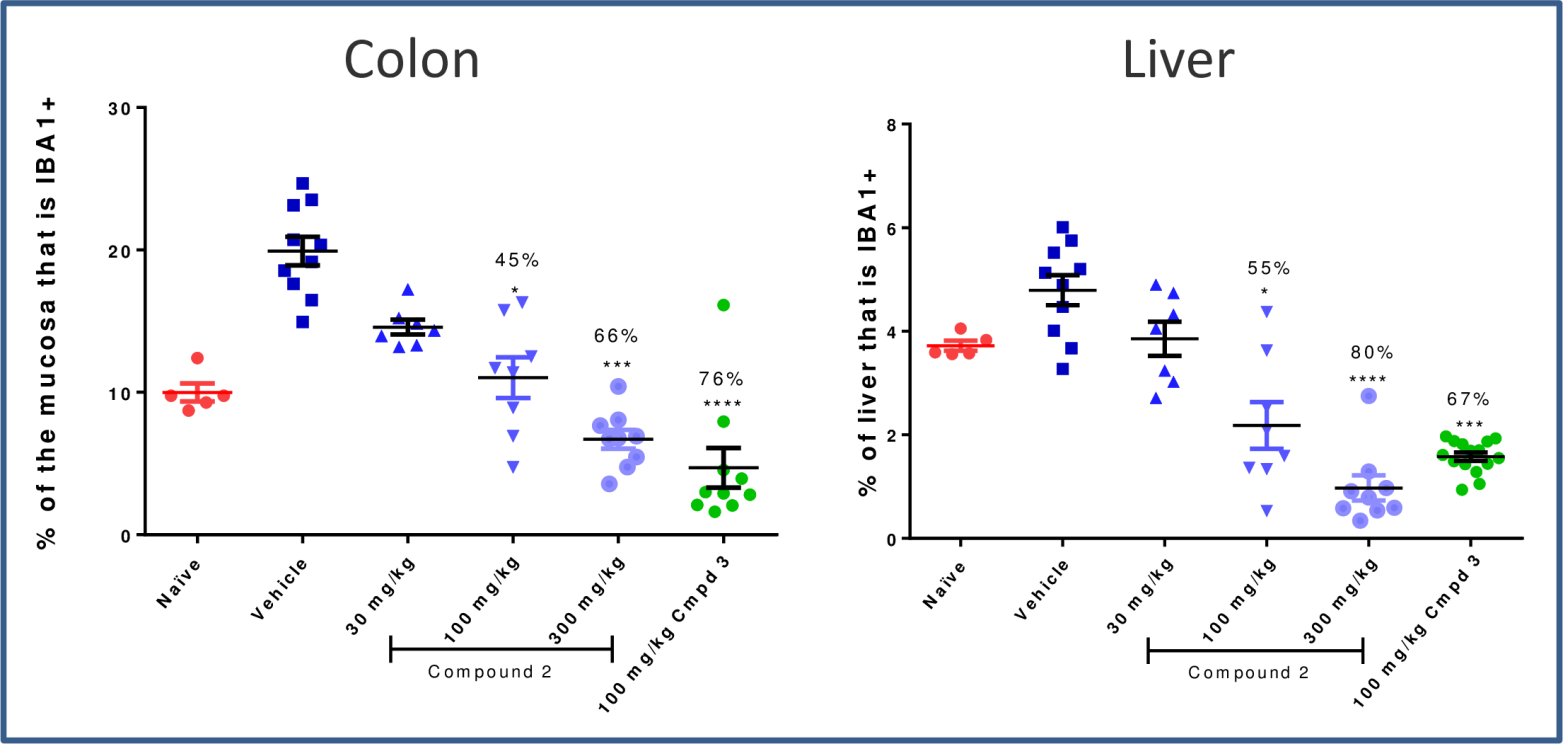
Depletion of macrophages in the colon and liver by image analysis. * p<0.05, *** p<0.001, ****p<0.0001; Kruskal-Wallis ANOVA with Dunn’s multiple comparison post-test vs. Vehicle. A subset of animals did not receive the final dose of **2** and were removed from the analysis.

**Fig 13:**
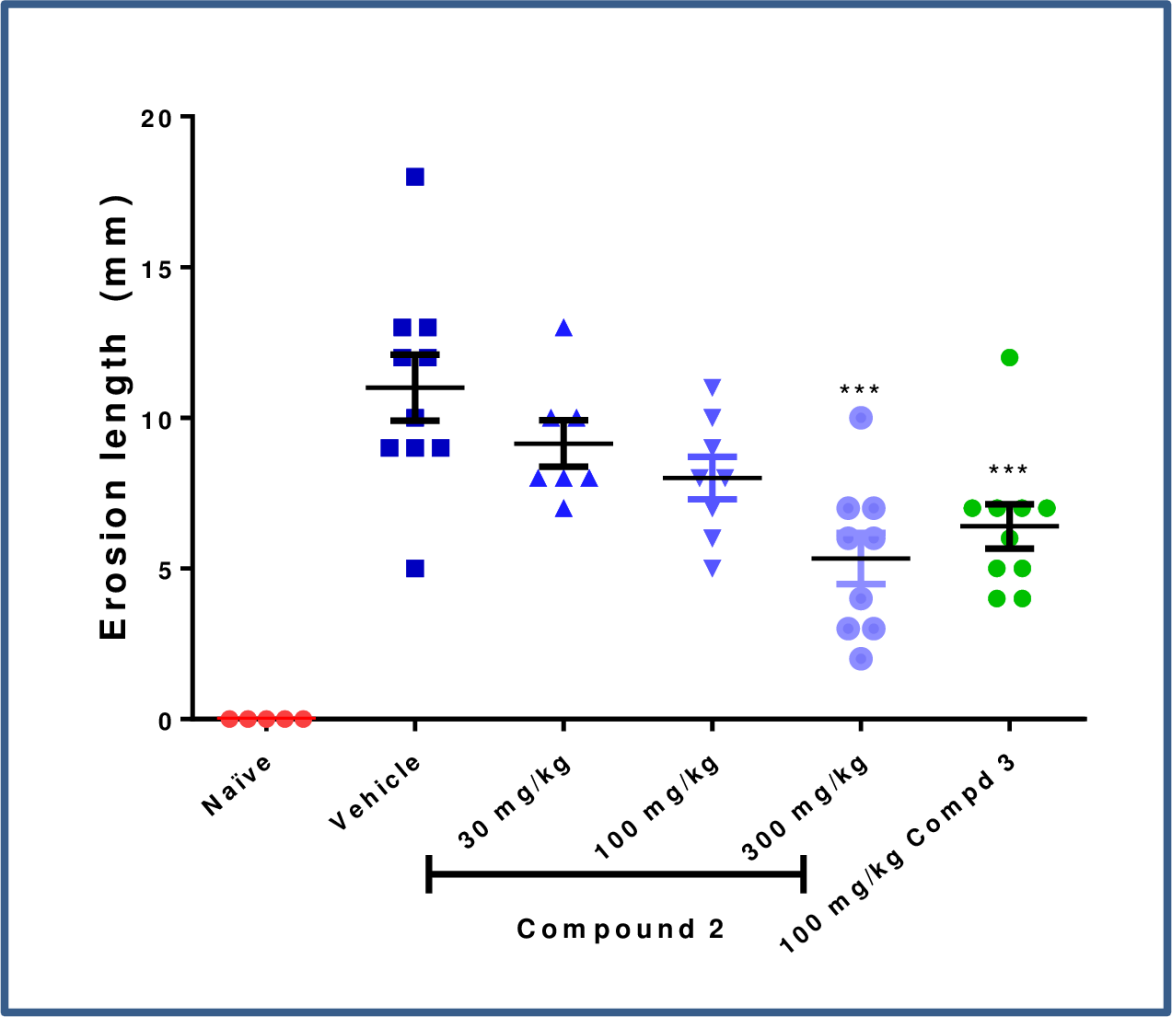
Change in colon erosion length by histology. *** p<0.001; One-way ANOVA with Dunnett’s multiple comparison post-test vs. Vehicle.

**Fig 14:**
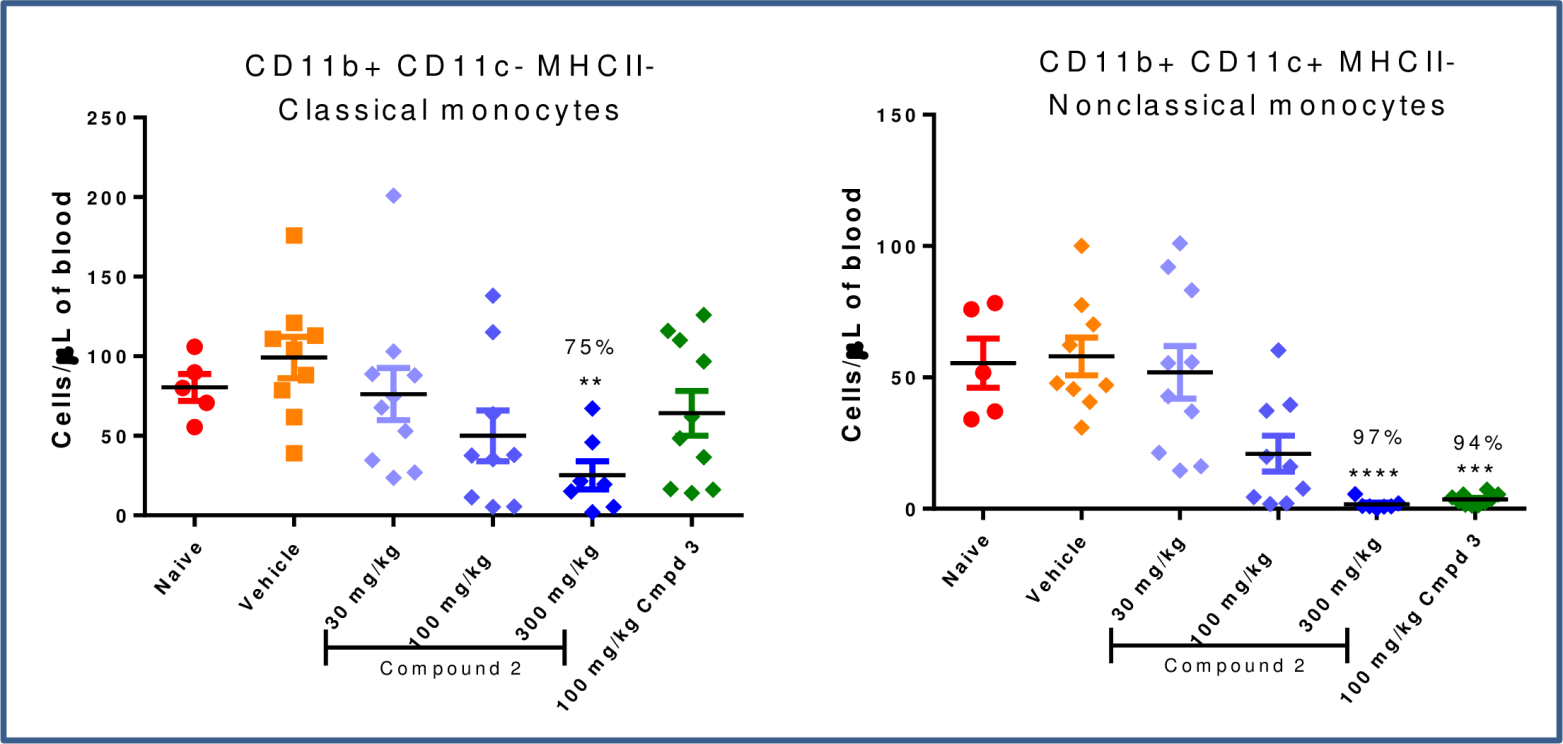
Depletion of circulating monocytes by FACS. ** p<0.01, *** p<0.001, **** p<0.0001; Kruskal-Wallis ANOVA with Dunn’s multiple comparison post-test vs. Vehicle.

At the lowest dose of prodrug tested (**2**, 30 mg/kg), a trend in reduction of IBA1+ cells was observed in the colon and the liver; however, this was not statistically significant (Fig 12). At higher doses, with concomitantly increasing systemic exposure, more substantial reductions in colon macrophages were observed, as were significant depletion of IBA1+ cells in the liver. The high dose (300 mg/kg) depleted colonic macrophages to a similar extent as systemic CSF1R inhibitor **3** (66% versus 76%, respectively). The impact of prodrug on colonic erosions was less significant, and a statistical decrease in erosion length with comparable effect to the positive control **3** was observed only at the 300 mg/kg dose (Fig 13). No significant effect on circulating CD11b+ CD11c- MHCII- monocytes nor on the more sensitive CD11b+ CD11c+ MHCII- monocytes was detected at the 30 or 100 mg/kg doses (Fig 14), which corresponded to the blood exposures below cellular EC_50_ at these doses. At the top dose (300 mg/kg), where the largest decrease in colonic macrophages was observed, there was nearly complete depletion of non-classical monocyte population to a level comparable to the effect with systemic inhibitor **3** (97% and 94%, respectively).

Overall, although colon-restricted drug exposure at the 30mpk dose was demonstrated, there was a lack of compelling efficacy on erosion length at this dose. Higher efficacy and more significant pharmacodynamic effects of IBA1 reduction in the colon were seen only at 100 and 300 mg/kg doses where significant IBA1 reduction was also observed in liver, signifying lack of a gut-restricted pharmacodynamic effect. If higher exposure in the colon could be achieved, while maintaining similar (or improved) colon:plasma or colon:liver exposures, it may be feasible to achieve a more substantial local effect, but the apparent sensitivity of circulating monocytes and liver IBA1+ cells to CSF1R inhibition made this approach significantly less attractive. Nonetheless, the ability to practically achieve high colonic and low systemic exposure by utilizing cyclodextrin prodrugs could be a promising approach for other biological targets that would benefit from colon-restricted delivery.

### Chemistry

**Fig 15:**
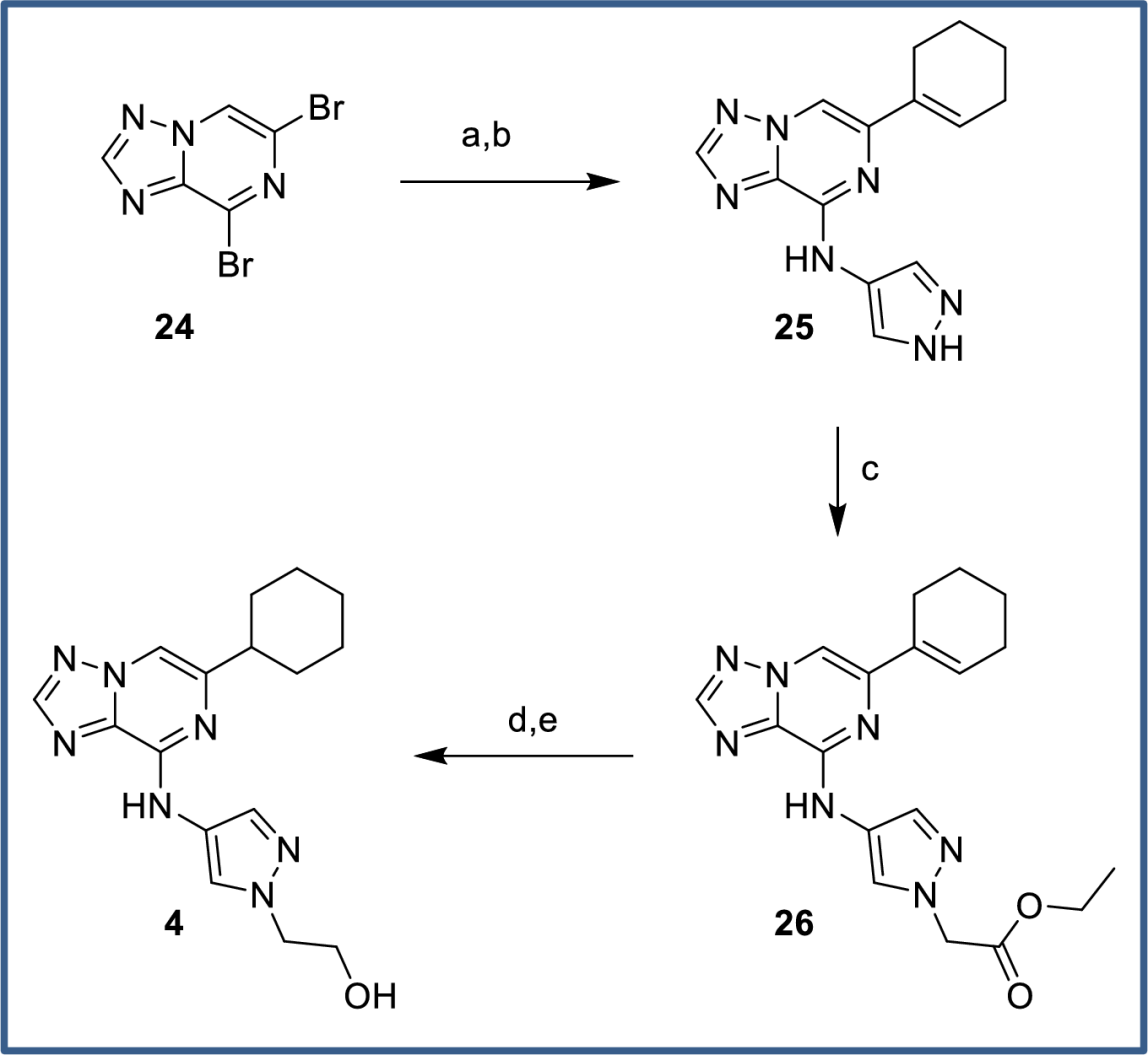
Synthesis of primary alcohol 4. Reagents and conditions: (a) 4-aminopyrazole hydrochloride, Et_3_N, DMF, 87%; (b) cyclohex-1-en-1-ylboronic acid, 2-(cyclohex-1-en-1-yl)-4,4,5,5-tetramethyl-1,3,2-dioxaborolane, PdCl_2_dppf, K_3_PO_4_, dioxane/water, 90°C, 46%; (c) ethyl 2-bromoacetate, K_2_CO_3_, DMF, 90°C, 45% (+ 31% recovered **25**); (d) 10% Pd/C, H_2_ (1 atm), MeOH/EtOAc, rt, 84%; (e) LiAlH_4_, THF, 0°C to rt, 75%.

The synthesis of primary alcohol CSF1R inhibitor **4** is shown in Fig 15. Displacement of bromide **24** with 4-aminopyrazole occurred with excellent regioselectivity at C8. We explored direct introduction of the cyclohexenyl substituent at C6 via Negishi coupling, however the yields were generally moderate and scalability was poor. Better overall yields were typically obtained by stepwise introduction of the cyclohexenyl substituent via Suzuki coupling and hydrogenation. Using this approach, cyclohexene **25** was prepared in moderate yield from the 6-bromo-8-aminopyrazolo-triazolopyrazine intermediate. The subsequent alkylation of the pyrazole with ethyl bromoacetate only partially went to completion but the product was readily separated from starting material **25** and underwent reduction of the alkene and ester functionalities to give primary alcohol **4**.

**Fig 16:**
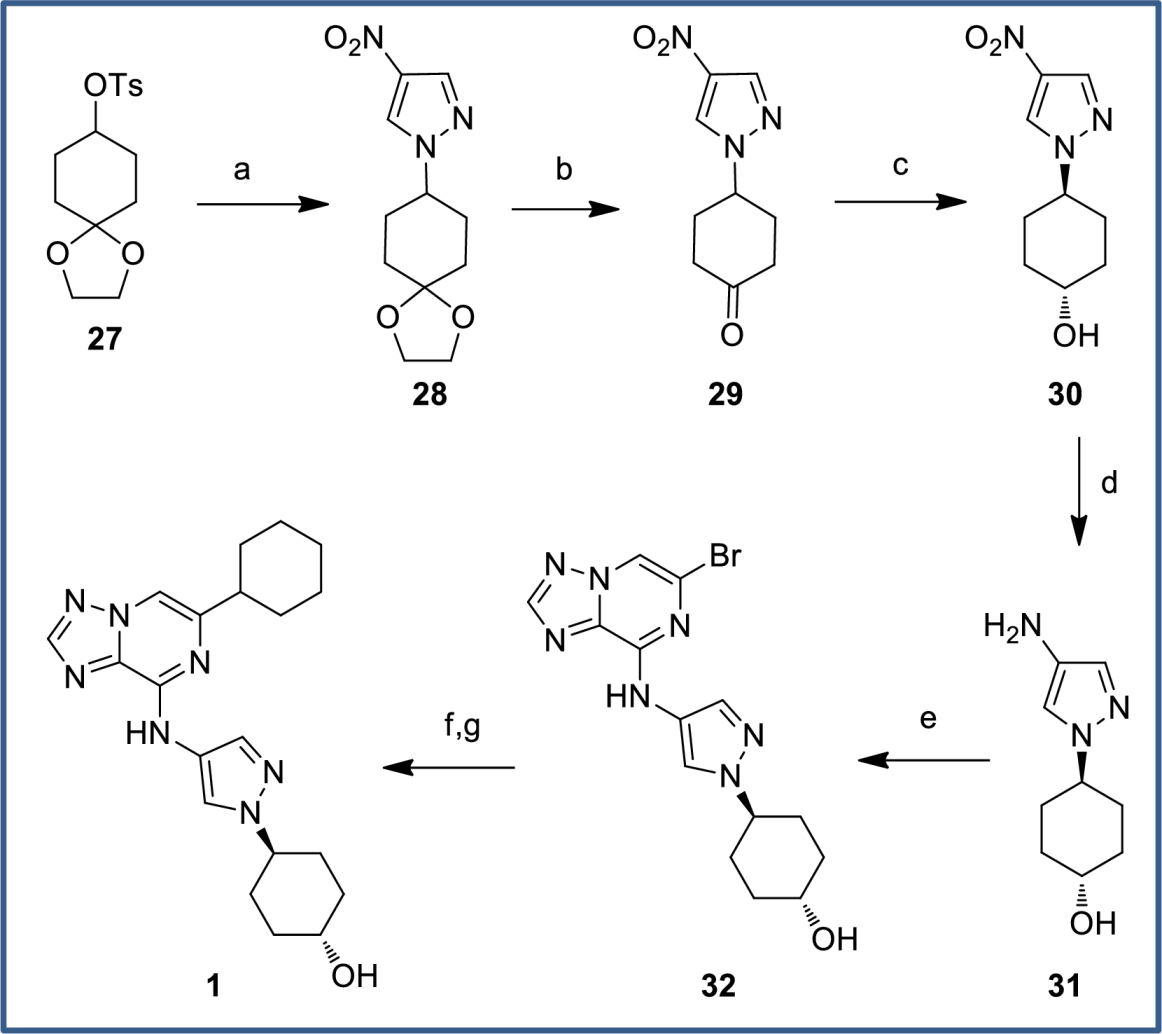
Synthesis of secondary alcohol 1. Reagents and conditions: (a) 4-nitropyrazole, Cs_2_CO_3_, DMF, 77% (b) PPTS, acetone/H_2_O, 88%; (c) NaBH_4_, EtOH, -5°C; recrystallization from toluene, 59%; (d) Pd/C, H_2_ (1 atm), MeOH, rt, 98%; (e) **24**, DIPEA, DMF, rt, 97%; (f) cyclohex-1-en-1-ylboronic acid, Pd(PPh_3_)_2_Cl_2_, Cs_2_CO_3_, 1,4-dioxane/water, 90°C, quant; (g) Pd(OH)_2_, H_2_ (1 atm), THF/MeOH/AcOH, rt, 85%.

An optimized route for the synthesis of secondary alcohol **1** is shown in Fig 16. We knew from earlier studies that separating *cis*- and *trans*-**1** alcohols at a late stage would be difficult. We therefore focused on a route that would allow for the relative stereochemistry to be set at an early stage, targeting nitropyrazole **30** as a key intermediate. Following literature precedent with the corresponding iodopyrazole,[48] 4-nitropyrazole smoothly displaced sulfonate **27** to give acetal **28** which was deprotected using acidic conditions to provide ketone **29**. Reduction with sodium borohydride gave a 4:1 mix of *trans*:*cis* alcohols, which could be recrystallized from toluene to furnish *trans*-alcohol **30** with greater than 99:1 *trans*:*cis* selectivity on greater than 40 g scale. Reduction of the nitro group cleanly yielded primary amine **31** which underwent regioselective S_N_Ar displacement at the C8 position of dibromide **24** to give bromide **32**. Final introduction of the cyclohexyl group was achieved by Suzuki coupling and hydrogenation of the resultant alkene to furnish secondary alcohol **1** in excellent yield.

**Fig 17:**
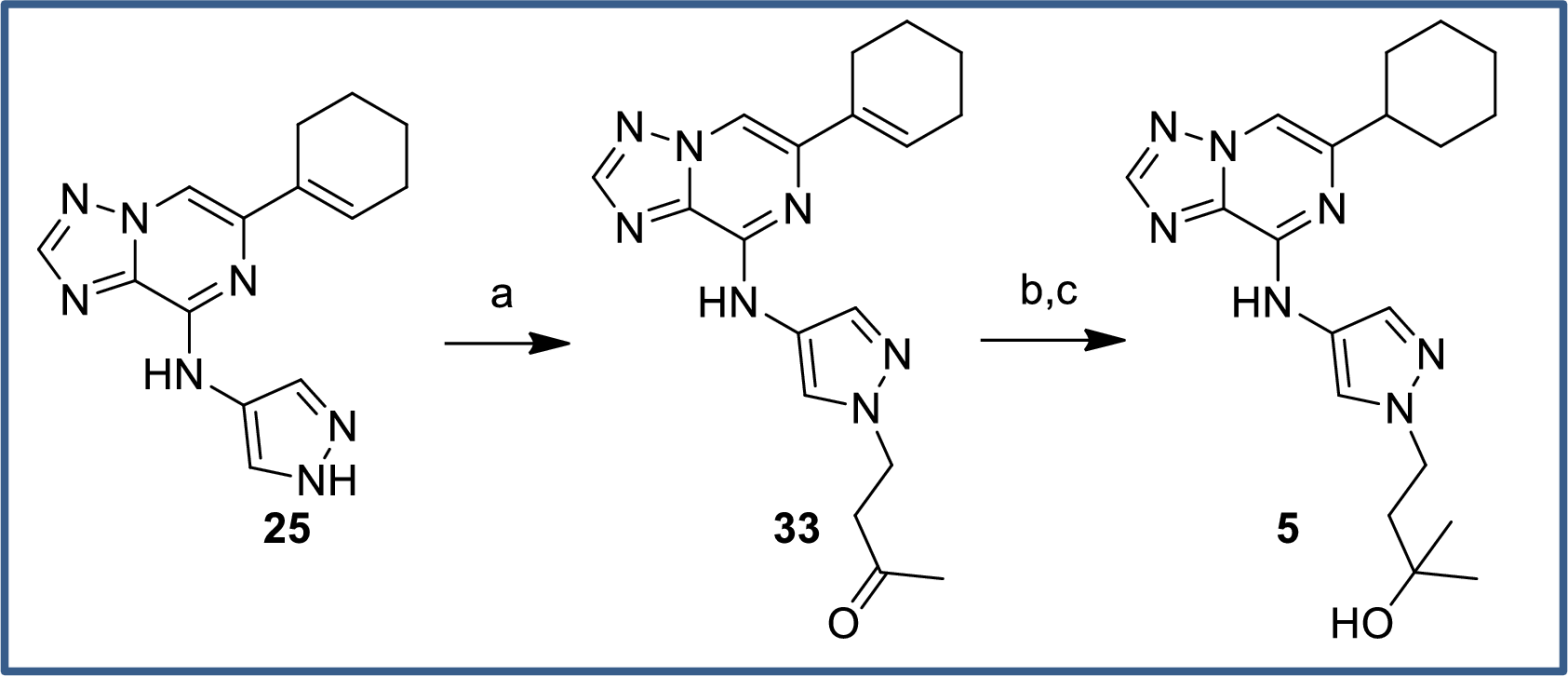
Synthesis of tertiary alcohol 5. Reagents and conditions: (a) but-3-en-2-one, Cs_2_CO_3_, DMF, rt, 73%; (b) MeMgBr, THF, rt, 38% (+ 44% recovered **33**); (c) Pd(OH)_2_, H_2_ (1 atm), MeOH/EtOAc/AcOH, rt, 62%.

Tertiary alcohol **5** was prepared from pyrazole intermediate **25** over three steps. Ketone intermediate **33** was accessed via a straight-forward Michael addition of pyrazole **25** with but-3-en-2-one. Addition of methylmagnesium bromide to the ketone moiety generated the tertiary alcohol in moderate yield, along with recovered starting material. Subsequent hydrogenation of the cyclohexene ring provided the desired tertiary alcohol **5** (Fig 17).

**Fig 18:**
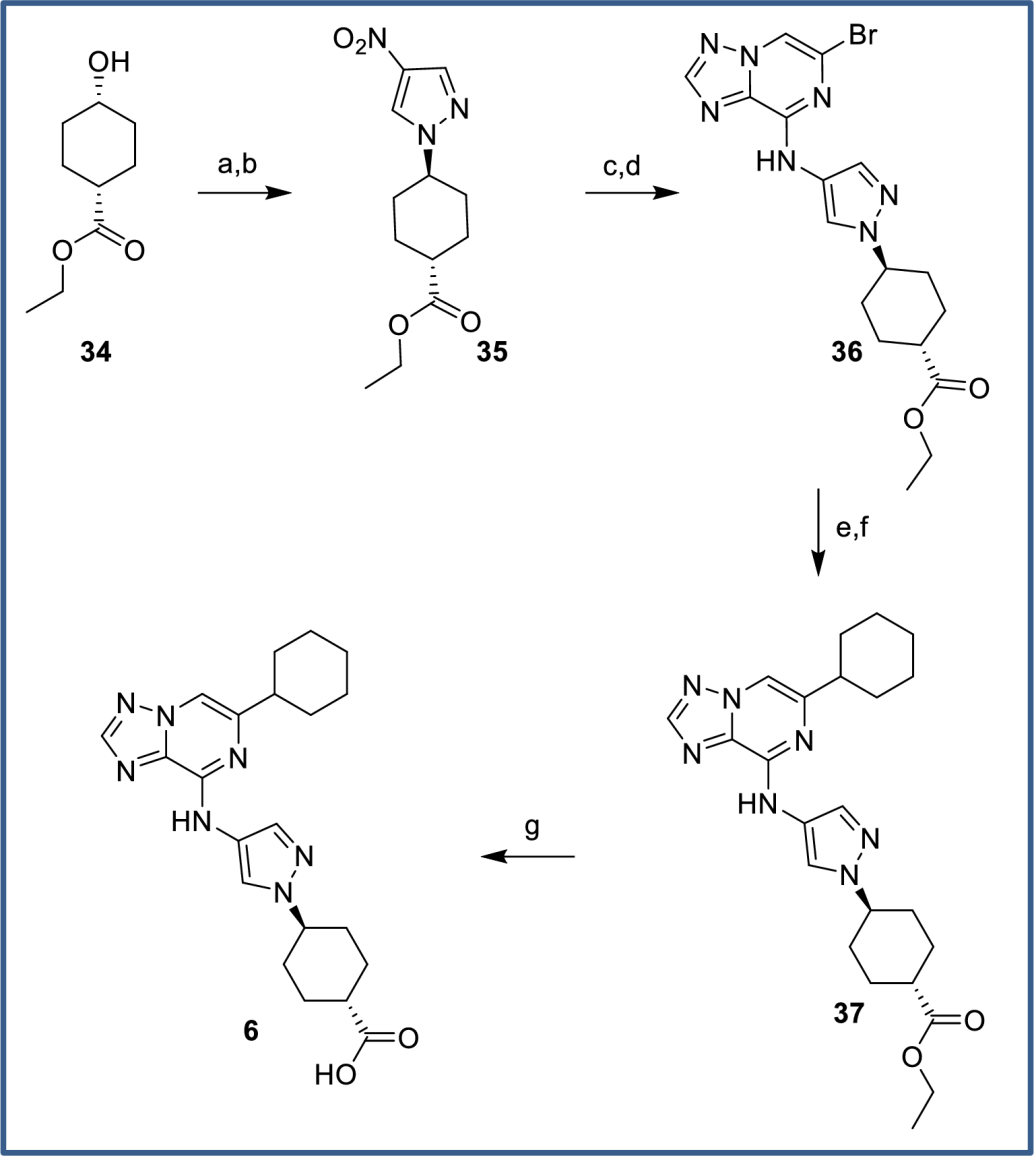
Synthesis of carboxylic acid 6. Reagents and conditions: (a) TsCl, pyridine, 0°C to rt, 80%; (b) 4-nitropyrazole, Cs_2_CO_3_, DMF, 77%; (c) 10% Pd/C, H_2_ (1 atm), THF/EtOH, rt, 93%; (d) **24**, Et_3_N, DMF, rt, 97%; (e) cyclohex-1-en-1-ylboronic acid, Pd(PPh_3_)_2_Cl_2_, Cs_2_CO_3_, 1,4-dioxane/water, 90°C, 89%; (f) Pd(OH)_2_, H_2_ (1 atm), MeOH/THF, rt, 87%; (g) NaOH, MeOH/H_2_O, rt, 96%.

Carboxylic acid **6** was prepared without incident via a closely related sequence (Fig 18). Conversion of commercially available *cis*-alcohol **34** to the corresponding tosylate followed by displacement with 4-nitropyrazole gave pyrazole **35**. Pd-catalyzed reduction of the nitro substituent and addition of the resulting aminopyrazole to C8 of dibromide **24** gave triazolopyrazine **36** as the only regioisomer. Suzuki coupling and hydrogenation introduced the cyclohexyl substituent, and a final saponification provided the desired acid **6**.

**Fig 19:**
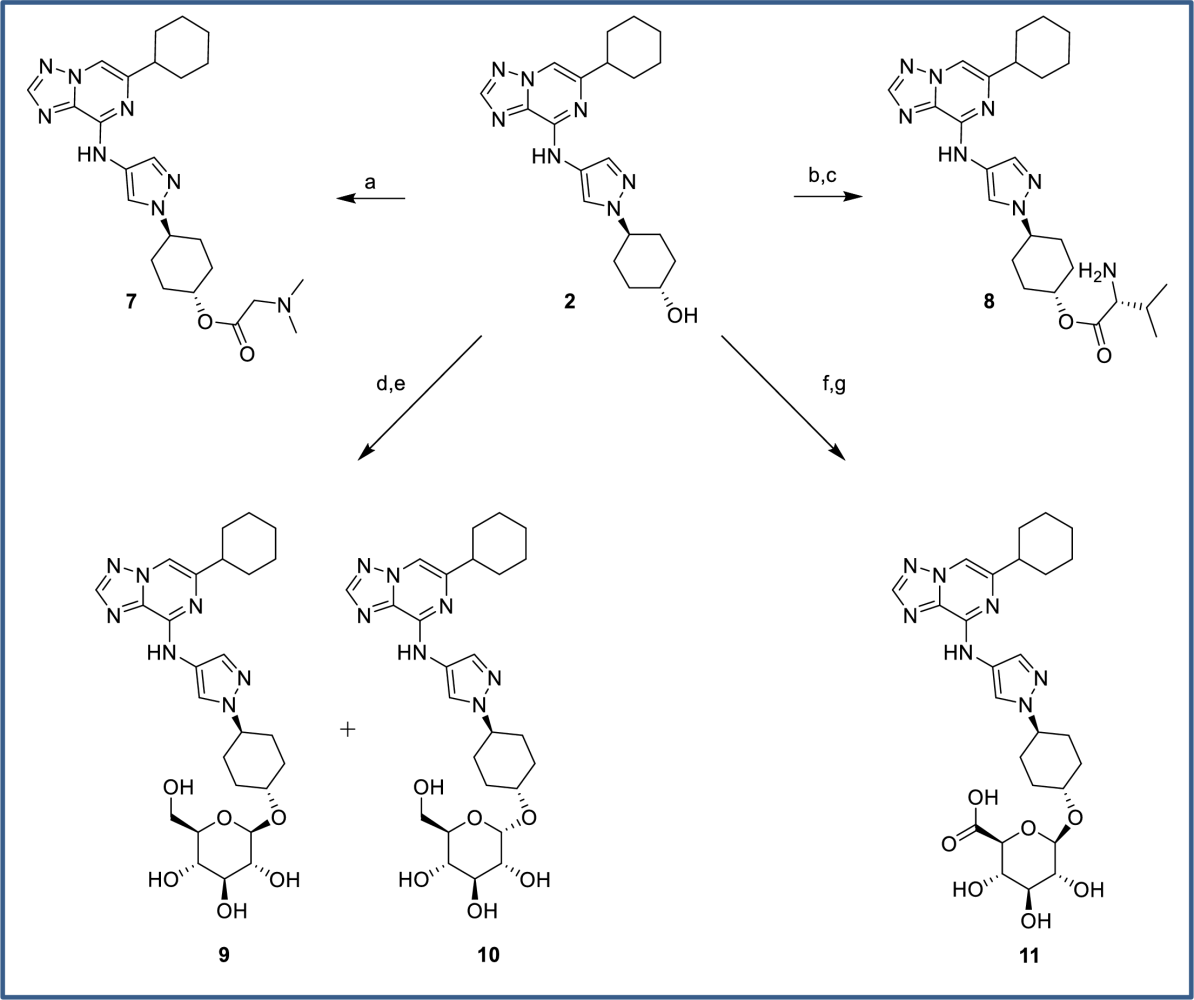
Synthesis of ester, glucoside and glucuronide prodrugs. Reagents and conditions: (a) 2-(dimethylamino)acetic acid, EDC, DMAP, DCM, rt, 92%; (b) Boc-(D)-valine, EDC, DMAP, DCM, rt, 72%; (c) TFA, DCM, 0°C to rt, 73% (d) 2,3,4,6-tetra-*O*-benzyl-alpha-D-glucopyranosyl trichloroacetimidate, BF_3_.OEt_2_, -78°C to rt, DCM, 95%; (e) Pd/C, H_2_ (10 bar), rt, 30% **9**, 17% **10**; (f) (2*R*,3*R*,4S,5*S*,6*S*)-2-bromo-6-(methoxycarbonyl)tetrahydro-2*H*-pyran-3,4,5-triyl triacetate, AgOTf, DCM, 5°C to rt, 7%; (g) LiOH, THF/H_2_O, 0°C, 50%.

Ester prodrugs **7** and **8** were prepared via straightforward coupling with EDC (Fig 19), followed by Boc-deprotection in the case of ester **8**. Preparation of glycosides **9** and **10** was initially attempted unsuccessfully via displacement of a tetrapivaloylglucopyranosyl bromide without success. In an alternative approach, Lewis acid mediated displacement of a tetrabenzyl trichloroacetimidate displacement proceeded to generate the protected glycosides excellent yield. Pd-catalyzed deprotection afforded anomeric products **9** and **10** which were readily separated by HPLC. Glucuronide **11** was prepared in low yield via displacement of a triacetyl 2-bromotetrahydropyran followed by base-catalyzed acetate deprotection. The poor yield was attributed to a competing acetyl transfer from the reagent to alcohol **2**.

**Fig 20:**
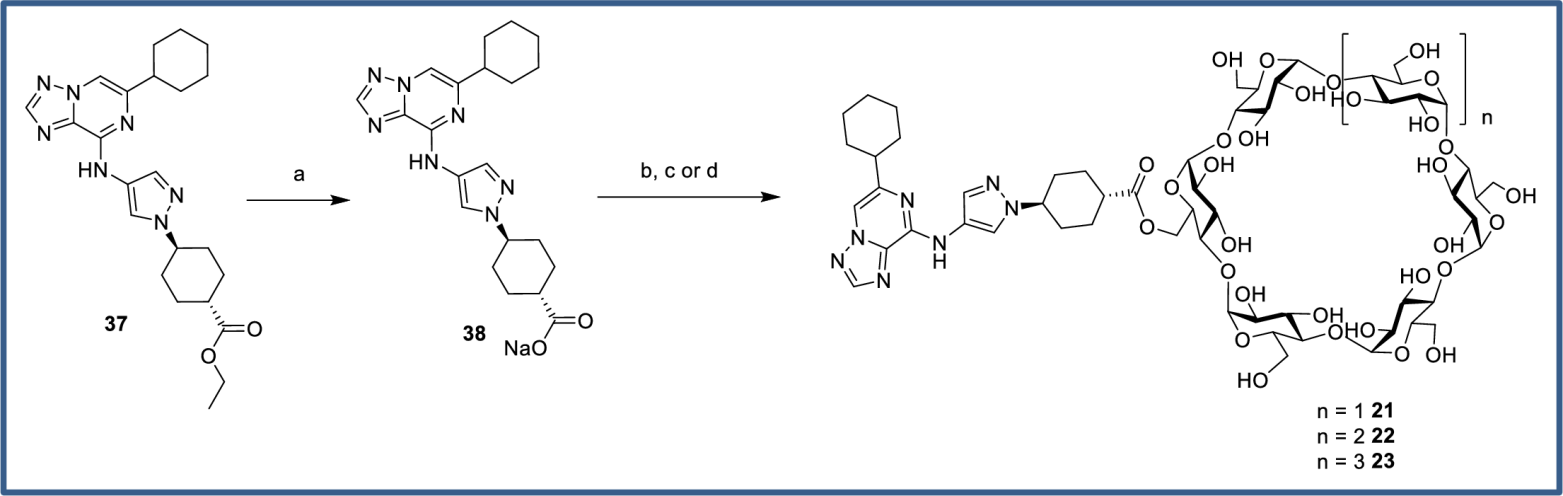
Synthesis of carboxylate-linked cyclodextrin prodrugs. Reagents and conditions: (a) NaOH, MeCN/H_2_O, quant; (b) Mono-6-*O*-(*p*-toluenesulfonyl)-α-cyclodextrin, DMA, 100°C, 33%; (c) Mono-6-*O*-(*p*-toluenesulfonyl)-β-cyclodextrin, DMA, 100°C, 23%; (d) Mono-6-*O*-mesitylenesulfonyl-γ-cyclodextrin, DMA, 100°C, 27%.

Cyclodextrin prodrugs of carboxylic acid **6** were obtained via saponification of ester **37**, isolation of the sodium salt **38** and displacement with commercially available 6-*O*-sulfonylcyclodextrins (Fig 20). The reactions each proceeded to approximately 50% conversion by LCMS, at which point the crude reaction mixtures were directly subjected to reverse-phase HPLC. The desired prodrugs **21–23** were isolated as single regioisomers in modest yields.

**Fig 21:**
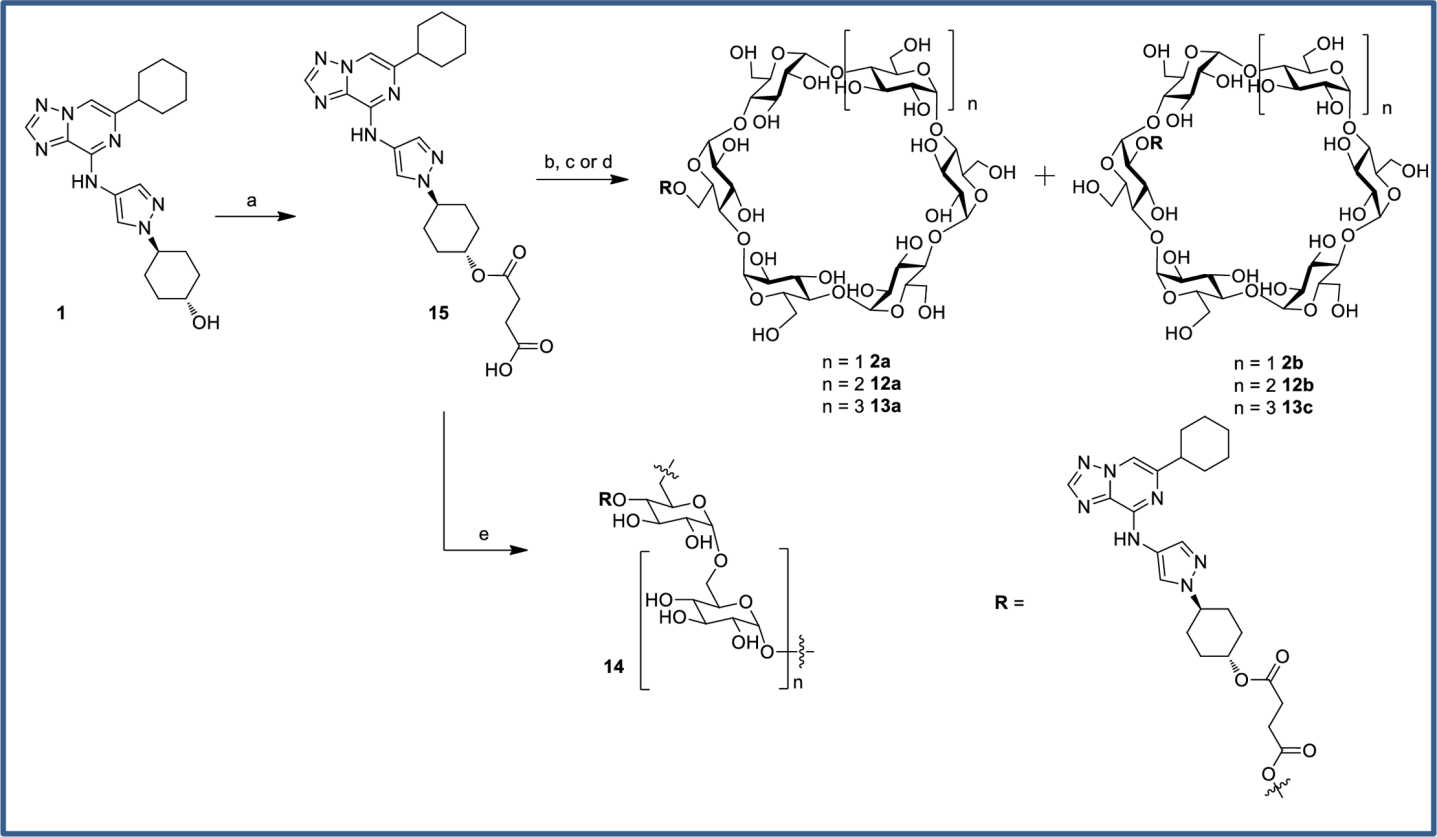
Synthesis of alcohol-linked cyclodextrin and dextran prodrugs. Reagents and conditions: (a) Et_3_N, succinic anhydride, DMAP, DCM, rt, 98%; (b) α-cyclodextrin, EDC, DMAP, rt, 32%, 82:18 mix of **2a**:**2b**; (c) β-cyclodextrin, EDC, DMAP, rt, 11%, 42:58 mix of **12a**:**12b**; (d) γ-cyclodextrin, EDC, DMAP, rt, 21% 70:30 mix of **13a**:**13b**; (e) CDI, Et_3_N, dextran (MW ~70,000) from *Leuconostoc* spp, DMSO, rt, 27%.

**Fig 22:**
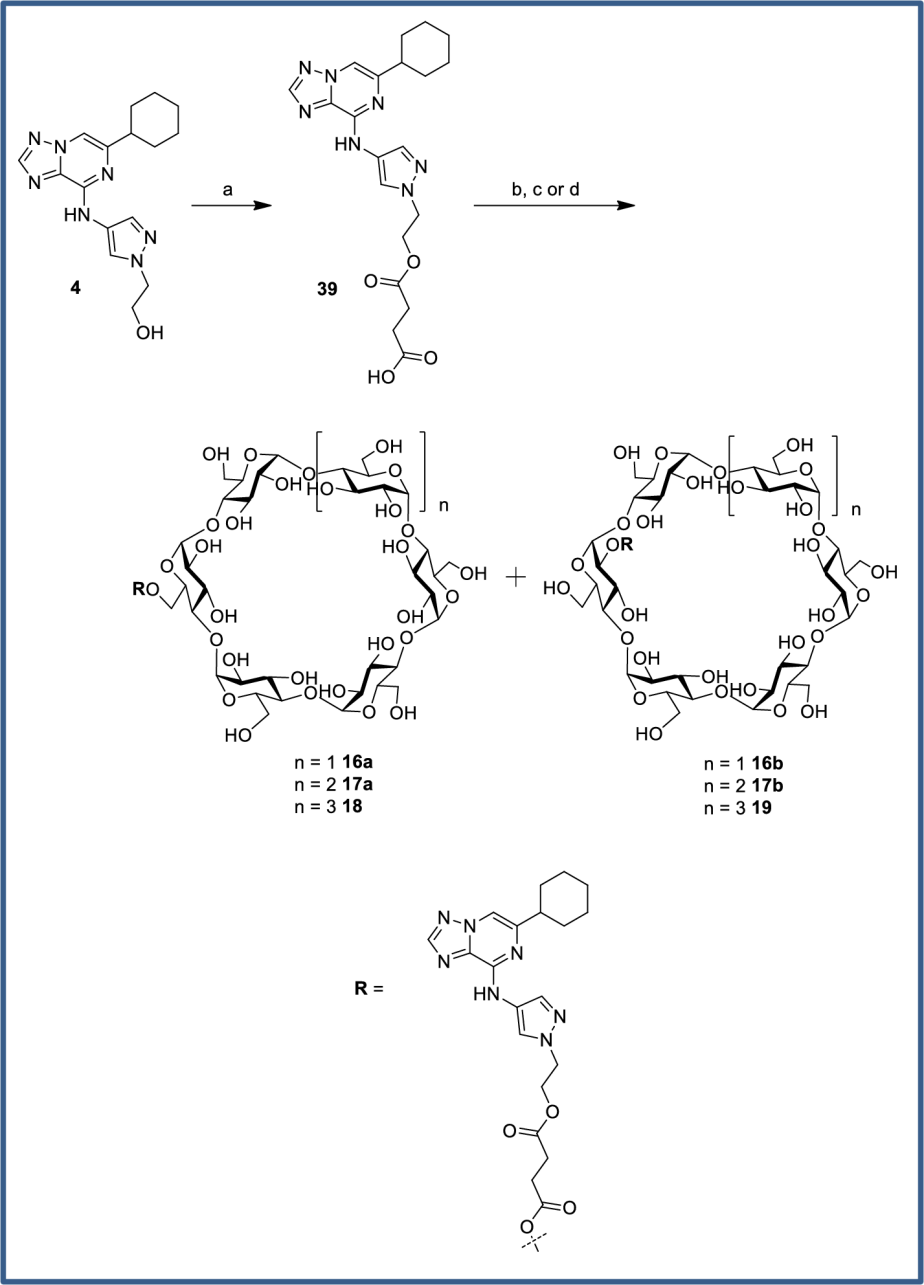
Chemical Synthesis of primary alcohol-linked cyclodextrin prodrugs. Reagents and conditions: (a) Et_3_N, succinic anhydride, DMAP, DCM, rt, 71%; (b) α-cyclodextrin, EDCI, DMAP, rt, 22%, 68:32 mix of **16a**:**16b**; (c) β-cyclodextrin, EDCI, DMAP, rt, 5.2% **17a** only; (d) γ-cyclodextrin, EDCI, DMAP, rt, 14% **18**, 17% **19**.

**Fig 23:**
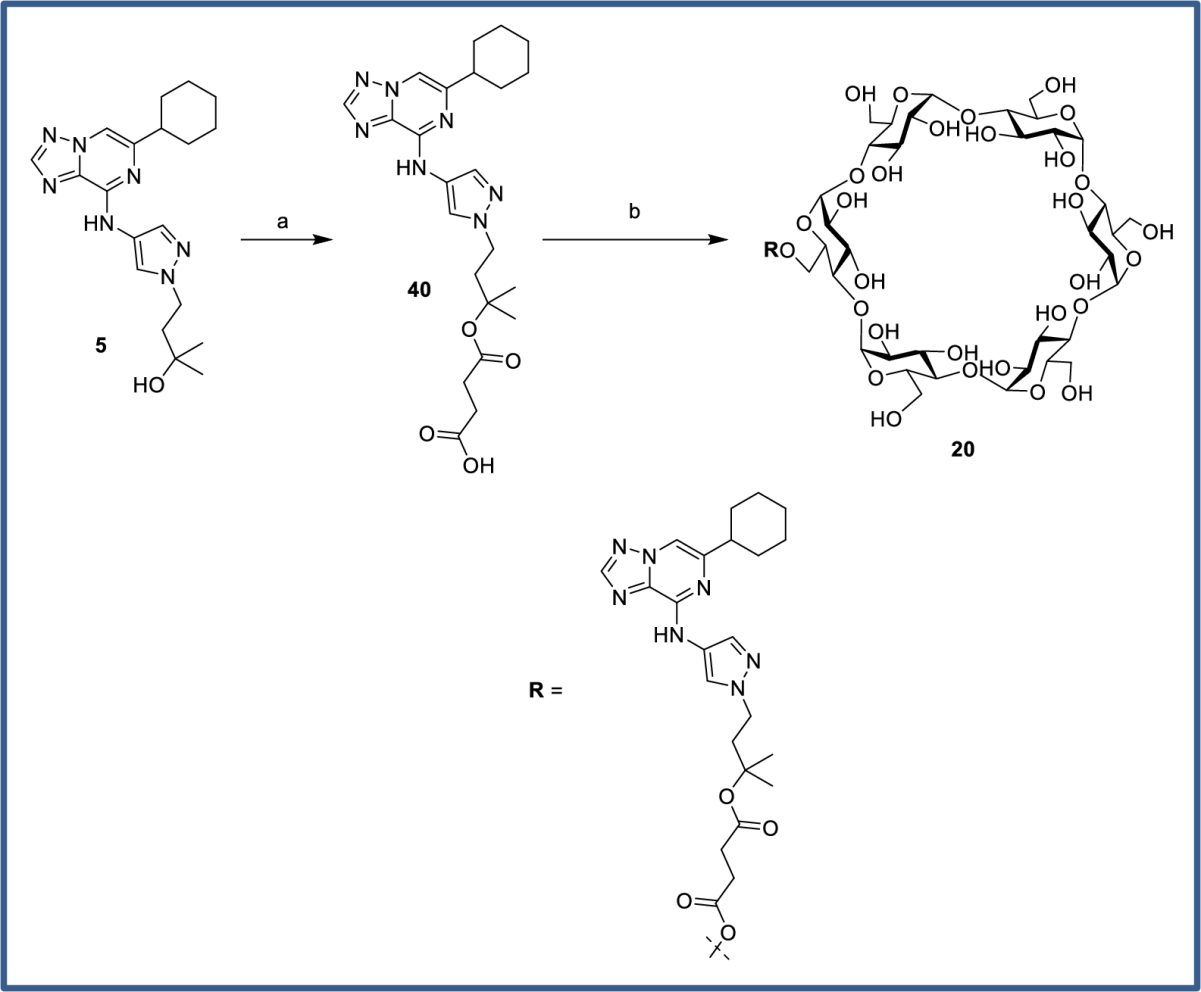
Chemical synthesis of tertiary alcohol-linked cyclodextrin prodrug. Reagents and conditions: (a) Et_3_N, succinic anhydride, DMAP, *N*-hydroxysuccinimide, toluene, 120°C, 54%; (b) α-cyclodextrin, EDCI, DMAP, rt, 22%.

Cyclodextrin conjugation of the primary alcohol was performed using a modification of the literature procedure for prednisolone.[49] Formation of hemisuccinate ester **15** via reaction with succinic anhydride (Fig 21) proceeded in excellent yield. In contrast to literature reports, CDI coupling with cyclodextrins were ineffective. Instead, EDC coupling with an excess of α, β or γ-cyclodextrin was used to provide the conjugates **2**, **12** and **13**. Precipitation with acetone gave solids which could be purified via reverse-phase HPLC purification, albeit as inseparable mixtures of 6-*O* and 2-*O* cyclodextrins. The poor yields were again attributable to incomplete conversion. The ratio of products was quantitated by ^1^H-NMR, with the triazole proton being the most clearly resolved peak despite being remote to the point of attachment. The regiochemistry was also determined by NMR, with a significant downfield shift observed for the cyclodextrin C-H protons at each point of attachment. The cyclodextrin prodrugs of primary alcohol **4** and tertiary alcohol **5** were prepared similarly (Fig 22 and Fig 23). The dextran conjugate of alcohol **1** was prepared using a protocol developed for dexamethasone.[50] Hemisuccinate **15** was treated with CDI followed by dextran (MW ~70,000) from *Leuconostoc* spp (Fig 21). After trituration of the resultant solid which removed any unconjugated starting material, the extent of substitution was determined via hydrolysis with aqueous NaOH and HPLC quantitation of the released parent. The solid was determined to contain 11.5 wt% **1**.

## Conclusions

Colon-restricted delivery has the potential to be a powerful tool for delivering efficacy for GI targets while reducing on-target or off-target side effects resulting from systemic exposure achieved by traditional oral delivery methods. Prodrugs could be of particular use as tools to generate preclinical *in vivo* proof-of-concept in mouse models of GI disease. We described the synthesis of a series of prodrugs appended to the same active parent molecule, and for each evaluated colon versus systemic exposure of active parent upon oral dosing of prodrug. Of a broad range of prodrugs evaluated, the cyclodextrin-conjugated analogs proved optimal for GI-restricted delivery when coupled to an active parent molecule with low–moderate absorption (FaFg). Carboxylic acids, primary alcohols, and secondary alcohols all serve as viable synthetic attachments for cyclodextrin appendage points, enabling the demonstration of restricted delivery to the colon, with up to >100x unbound colon:plasma exposure ratios. Optimal synthetic routes to synthesize cyclodextrin prodrugs were developed and the secondary alcohol α-cyclodextrin derivative **2** was prepared on multi-gram scale to enable *in vivo* proof-of-concept experiments. In a mouse colitis model, target colon exposures with minimal systemic exposure were successfully achieved with **2** when dosed at 30 mg/kg (active parent-dose equivalent) BID, however only a moderate pharmacodynamic effect, measured as a reduction of IBA1+ macrophages in the colon, was observed. Optimal depletion of colon macrophages was achieved only at higher doses, and these doses resulted in attendant systemic exposure, depletion of circulating monocytes, and reduction of liver macrophages.

Although an increased colon:systemic exposure margin was achieved, the DSS colitis model results suggest a gut-restricted CSF1R inhibitor is only partially effective. This is likely tied to the biology of monocyte recruitment from the blood to the gut both in healthy and inflamed settings, suggesting that systemic CSF1R inhibition is required to elicit full colon macrophage depletion and colitis disease reduction, and perhaps CSF1R was not an ideal target to test proof-of-concept for gut-delivery strategies.

Although CSF1R protein expression is mainly discussed in literature as linked to myeloid lineage cells, there are reports of receptor expression in gut epithelial cells.[51, 52] Upon investigation of CSF1R expression patterns by immunohistochemistry (IHC) in mouse and human colon tissue, our group was unable to corroborate CSF1R epithelial staining patterns, observing only myeloid/macrophage cell staining with little to no epithelial cell staining. To note, the differences in IHC expression observed by our group and others could be attributed to different anti-CSF1R monoclonal antibody clones used for staining. A more comprehensive evaluation assessing CSF1R expression in multiple tissues and species would be needed to better understand the role of CSF1R inhibition in modulating gut epithelium homeostatic mechanisms.

On the whole, the pro-drug strategies and learnings employed herein may be better-suited for alternative targets of GI-disease, and these efforts have at least provided a comprehensive frame-work in which to pursue additional gut-restricted delivery strategies for future GI targets.

## Materials and methods

### General methods

All reagents and anhydrous solvents were obtained from commercial sources and used without further purification unless noted otherwise. All reactions involving air- or moisture-sensitive reagents were performed under a nitrogen atmosphere. ^1^H-NMR spectra were obtained on an Agilent 400-MR DD2 with ONE_NMR probe actively shielded 400 MHz at 30°C. All final compounds were purified to ≥95% purity unless otherwise noted, as determined by HPLC-MS obtained on a Waters Acquity UPLC instrument using either [A] 2.1 x 30 mm Waters Cortecs C18 column (1.6 μm particles). [B] 2.1 x 30 mm Halo-2 C8 column (2μm particles). [C] 4.6 x 50 mm MAC-MOD Halo C8 column (2.7 μm particles). [D] 4.6 x 50 mm MAC-MOD Halo C18 column (2.7 μm particles). Detection methods were diode array (DAD) and evaporative light scattering (ELSD) detection as well as positive/negative electrospray ionization. Preparative HPLC-MS was performed on a Waters AutoPurification system using either [A] Phenomenex: Synergi MaxRP Prep, 4 μm particle size 19 x 150 mm column [B] Waters: Atlantis Prep T3 OBD, 5 μm particle size 50 x 100mm column [C] Phenomenex: Synergi Polar-RP 80A, 4 μm particle size 21.2 x 150 mm column.

#### 6-(Cyclohex-1-en-1-yl)-N-(1*H*-pyrazol-4-yl)-[1,2,4]triazolo[1,5-*a*]pyrazin-8-amine (25)

6,8-dibromo-[1,2,4]triazolo[1,5-*a*]pyrazine (22.1 g, 80 mmol) and 1*H*-pyrazol-4-amine hydrochloride (10.0 g, 84 mmol) were combined in DMF (398 mL). Triethylamine (33.3 mL, 239 mmol) was added and the mixture was heated to 95°C for 18 h. The reaction mixture was then cooled to ambient temperature and concentrated under reduced pressure. The residue was suspended in water (300 mL) and sonicated and filtered. Trituration in ether provided 6-bromo-*N*-(1*H*-pyrazol-4-yl)-[1,2,4]triazolo[1,5-a]pyrazin-8-amine (19.3 g, 68.9 mmol, 87%); ^1^H NMR (400 MHz, DMSO-*d*_6_) δ 12.67 (s, 1H), 10.65 (s, 1H), 8.54 (s, 1H), 8.10 (s, 1H), 7.82 (s, 1H).

A flask was charged with 6-bromo-*N*-(1*H*-pyrazol-4-yl)-[1,2,4]triazolo[1,5-*a*]pyrazin-8-amine (4.40 g, 15.7 mmol), 2-(cyclohex-1-en-1-yl)-4,4,5,5-tetramethyl-1,3,2-dioxaborolane (5.23 g, 25.1 mmol), PdCl_2_(dppf) (1.149 g, 1.571 mmol) and potassium phosphate (8.34 g, 39.3 mmol). 1,4-dioxane (60 mL) and water (15 mL) were added. The mixture was sparged with nitrogen for 5 min and heated at 90°C for 16 h. The reaction mixture was cooled and charged with cyclohex-1-en-1-ylboronic acid (0.989 g, 7.85 mmol) and PdCl_2_(dppf) (0.575 g, 0.785 mmol). The flask was degassed and then stirred at 90°C overnight. The mixture was diluted with water (200 mL) and filtered. The precipitate was stirred in MeOH and filtered again. The precipitate was kept aside. The filtrate was concentrated and the residue was suspended in MeOH and filtered to give an additional batch of precipitate. The two batches were combined and triturated with MeOH:AcOH:THF (4:1:1) to give the title compound (2.02 g, 46%); ^1^H NMR (400 MHz, DMSO-*d*_6_) δ 10.13 (s, 1H), 8.49 (s, 1H), 8.15 (s, 1H), 8.01 (s, 2H), 6.85 (td, *J* = 3.9, 1.9 Hz, 1H), 2.41 (td, *J* = 6.1, 2.3 Hz, 2H), 2.26 (qd, *J* = 5.8, 3.7, 3.3 Hz, 2H), 1.78–1.57 (m, 4H).

#### Ethyl 2-(4-((6-(cyclohex-1-en-1-yl)-[1,2,4]triazolo[1,5-*a*]pyrazin-8-yl)amino)-1*H*-pyrazol-1-yl)acetate (26)

A suspension of 6-(cyclohex-1-en-1-yl)-*N*-(1*H*-pyrazol-4-yl)-[1,2,4]triazolo[1,5-*a*]pyrazin-8-amine (2.27 g, 8.07 mmol) and potassium carbonate (1.67 g, 12.1 mmol) in DMF (40 mL) was heated at 90°C for 2 min. Ethyl-2-bromoacetate (0.982 mL, 8.88 mmol) was added and the mixture was heated at 90°C for 3.5 h. The reaction mixture was partitioned between water (60 mL) and EtOAc (80 mL). The organic layer was dried over MgSO_4_, filtered, concentrated. The residue was washed with 10% MeOH/DCM. The precipitate was discarded and the filtrate was concentrated under reduced pressure. The residue was purified via flash chromatography (20–80% EtOAc/DCM) to give the title compound (1.34 g, 45%); MS *m/z*: 368 (M+H)^+^.

#### 2-(4-((6-Cyclohexyl-[1,2,4]triazolo[1,5-*a*]pyrazin-8-yl)amino)-1*H*-pyrazol-1-yl)ethanol (4)

A flask was charged with 10% Pd/C (1.51 g, 1.42 mmol) and ethyl 2-(4-((6-(cyclohex-1-en-1-yl)-[1,2,4]triazolo[1,5-*a*]pyrazin-8-yl)amino)-1*H*-pyrazol-1-yl)acetate (1.3 g, 3.54 mmol). The flask was maintained under vacuum and MeOH (20 mL) and EtOAc (20 mL) were added. The suspension was then flushed with nitrogen, degassed, and stirred at ambient temperature under an atmosphere of hydrogen for 16 h. The reaction mixture was filtered through Celite® and the filtrate was concentrated under reduced pressure to give ethyl 2-(4-((6-cyclohexyl-[1,2,4]triazolo[1,5-*a*]pyrazin-8-yl)amino)-1*H*-pyrazol-1-yl)acetate (1.10 g, 84%); MS *m/z*: 370 (M+H)^+^. ^1^H NMR (400 MHz, DMSO-*d*_6_) δ 10.16 (s, 1H), 8.46 (s, 1H), 8.17 (d, *J* = 0.6 Hz, 1H), 8.03 (d, *J* = 0.5 Hz, 1H), 7.87 (d, *J* = 0.6 Hz, 1H), 5.05 (s, 2H), 4.13 (q, *J* = 7.1 Hz, 2H), 2.59 (tt, *J* = 11.8, 3.6 Hz, 1H), 1.89 (d, *J* = 12.4 Hz, 2H), 1.79 (d, *J* = 12.8 Hz, 2H), 1.70 (d, *J* = 12.4 Hz, 1H), 1.56 (qd, *J* = 12.4, 3.2 Hz, 2H), 1.44–1.22 (m, 3H), 1.22–1.11 (m, 3H).

To a solution of ethyl 2-(4-((6-cyclohexyl-[1,2,4]triazolo[1,5-*a*]pyrazin-8-yl)amino)-1*H*-pyrazol-1-yl)acetate (1.00 g, 2.71 mmol) in THF (2 mL) at 0°C, lithium aluminum hydride (1M in THF, 5.41 mL, 5.41 mmol) was added dropwise. The reaction mixture was warmed to ambient temperature and stirred for 3 h. The mixture was then cooled to 0°C and quenched with a solution of saturated aqueous Rochelle's salt (50 mL), diluted with water (40 mL) and extracted with EtOAc (3 x 50 mL). The combined organic layers were dried over MgSO_4_, filtered and concentrated under reduced pressure. The resultant solid was triturated with DCM to give the title product. (0.664 g, 75%); MS *m/z*: 328 (M+H)^+^. ^1^H NMR (400 MHz, DMSO-*d*_6_) δ 10.10 (s, 1H), 8.45 (s, 1H), 8.17 (d, *J* = 0.8 Hz, 1H), 8.01 (d, *J* = 0.5 Hz, 1H), 7.83 (d, *J* = 0.7 Hz, 1H), 4.85 (t, *J* = 5.3 Hz, 1H), 4.11 (t, *J* = 5.6 Hz, 2H), 3.71 (q, *J* = 5.5 Hz, 2H), 2.58 (tt, *J* = 11.7, 3.4 Hz, 1H), 1.94–1.66 (m, 5H), 1.58 (qd, *J* = 12.3, 3.2 Hz, 2H), 1.43–1.18 (m, 3H).

#### 4-Nitro-1-(1,4-dioxaspiro[4.5]decan-8-yl)-1*H*-pyrazole (28)

A flask was charged with 1,4-dioxaspiro[4.5]decan-8-yl 4-methylbenzenesulfonate (176 g, 562 mmol) and DMF (700 mL). 4-Nitro-1*H*-pyrazole (60.5 g, 535 mmol) and Cs_2_CO_3_ (209 g, 642 mmol) were added and the mixture was heated to 100°C for 2 h. The reaction mixture was cooled to ambient temperature and poured into water (800 mL). The suspension was stirred at 0°C for 30 min and filtered. The precipitate was washed with water (500 mL) and dried to give the title compound (104 g, 77%); MS *m/z*: 254(M+H)^+^. ^1^H NMR (400 MHz, DMSO-*d*_6_) δ 8.92–8.82 (m, 1H), 8.24 (d, *J* = 0.7 Hz, 1H), 4.42–4.27 (m, 1H), 3.92–3.79 (m, 4H), 2.00 (ddt, *J* = 8.8, 5.1, 3.4 Hz, 4H), 1.76 (ddt, *J* = 11.2, 3.8, 2.0 Hz, 2H), 1.72–1.58 (m, 2H).

#### 4-(4-Nitro-1*H*-pyrazol-1-yl)cyclohexanone (29)

A flask equipped with a mechanical stirrer was charged with 4-nitro-1-(1,4-dioxaspiro[4.5]decan-8-yl)-1*H*-pyrazole (104 g, 411 mmol), acetone (1027 mL) and water (1027 mL). Pyridine 4-methylbenzenesulfonate (211 g, 838 mmol) was added. The reaction mixture was heated to 65°C and stirred for 16 h. The reaction mixture was cooled to ambient temperature and concentrated to approximately half volume. A white solid precipitated and was collected via filtration. The collected solid was washed with water (700 mL) and dried at 60°C to yield the title compound (76.0 g, 88%); ^1^H NMR (400 MHz, DMSO-*d*_6_) δ 8.96 (d, *J* = 0.7 Hz, 1H), 8.28 (d, *J* = 0.7 Hz, 1H), 4.79 (tt, *J* = 10.5, 4.0 Hz, 1H), 2.67–2.52 (m, 2H), 2.37–2.16 (m, 6H).

#### (1r,4r)-4-(4-Nitro-1H-pyrazol-1-yl)cyclohexanol (30)

A mixture of 4-(4-nitro-1*H*-pyrazol-1-yl)cyclohexanone (74.5 g, 356 mmol) in ethanol (1.2 L) was cooled to -10°C. NaBH_4_ (3.50 g, 93 mmol) was added portion-wise, maintaining the temperature below -3°C. The reaction mixture was stirred for 40 min at -5°C then concentrated to 400 mL volume. To this mixture was added EtOAc (400 mL) and water (500 mL). The combined mixture was concentrated under reduced pressure to ~400 mL volume. The solution obtained was diluted with additional EtOAc (400 mL) and water (400 mL). The layers were separated. The aqueous layer was re-extracted with 10% MeOH/EtOAc (3 x 150 mL). The combined organic layers were dried over MgSO_4_, filtered and concentrated to give an off-white solid, (4:1 ratio of *trans*:*cis*; 72 g, 322 mmol, 96%). Recrystallization from hot toluene provided the title compound (44.5 g, 65%); ^1^H NMR (400 MHz, DMSO-*d*_6_) δ 8.85 (d, *J* = 0.7 Hz, 1H), 8.22 (d, *J* = 0.7 Hz, 1H), 4.65 (d, J = 4.4 Hz, 1H), 4.21 (tt, *J* = 11.7, 4.0 Hz, 1H), 3.46 (tq, *J* = 10.8, 4.3 Hz, 1H), 2.06–1.70 (m, 6H), 1.31 (tdd, *J* = 12.9, 10.7, 3.5 Hz, 2H).

#### (1*r*,4*r*)-4-(4-Amino-1*H*-pyrazol-1-yl)cyclohexanol (31)

10% Pd/C (9.96 g, 9.36 mmol) was added to a flask under an atmosphere of nitrogen. To this flask was added a solution of (1*r*,4*r*)-4-(4-nitro-1*H*-pyrazol-1-yl)cyclohexanol (49.4 g, 234 mmol) in MeOH (1 L) under vacuum. The reaction mixture was evacuated and purged with nitrogen 3 times then evacuated and stirred under hydrogen (1 atm) for 4 h. The reaction mixture was then evacuated and purged with nitrogen. The reaction mixture was filtered through Celite® and washed with MeOH (4 L) and EtOAc (1 L). The filtrate was concentrated under reduced pressure to give the title compound (41.7 g, 98%); ^1^H NMR (400 MHz, DMSO-*d*_6_) δ 6.99 (d, *J* = 0.9 Hz, 1H), 6.85 (d, *J* = 0.8 Hz, 1H), 4.56 (d, *J* = 4.4 Hz, 1H), 3.87 (tt, *J* = 11.7, 3.7 Hz, 1H), 3.68 (s, 2H), 3.43 (tq, *J* = 10.8, 4.1 Hz, 1H), 1.94–1.79 (m, 4H), 1.73–1.57 (m, 2H), 1.27 (tdd, *J* = 12.3, 10.8, 3.9 Hz, 2H). MS *m/z*: 182 (M+H)^+^.

#### (1r,4r)-4-(4-((6-Bromo-[1,2,4]triazolo[1,5-*a*]pyrazin-8-yl)amino)-1*H*-pyrazol-1-yl)cyclohexanol (32)

A flask equipped with a mechanical stirrer was charged with 6,8-dibromo-[1,2,4]triazolo[1,5-*a*]pyrazine (63.9 g, 230 mmol) and DMF (700 mL) followed by (1*r*,4*r*)-4-(4-amino-1*H*-pyrazol-1-yl)cyclohexanol (41.7 g, 230 mmol) and *N*-ethyl-*N*-isopropylpropan-2-amine (80 mL, 460 mmol). The reaction mixture was stirred at ambient temperature for 16 h. The reaction mixture was diluted with water (1.5 L) and filtered. The collected solid was triturated with water to give the title compound (84.0 g, 97%); MS *m/z*: 378,380 (M+H)^+^; ^1^H NMR (400 MHz, DMSO-*d*_6_) δ 10.63 (s, 1H), 8.54 (d, *J* = 5.6 Hz, 1H), 8.07 (s, 1H), 7.76 (s, 1H), 4.61 (d, *J* = 4.3 Hz, 1H), 4.20–4.05 (m, 1H), 3.48 (dq, *J* = 10.7, 5.6, 5.1 Hz, 1H), 2.08–1.85 (m, 4H), 1.85–1.64 (m, 2H), 1.43–1.19 (m, 2H).

#### (1*r*,4*r*)-4-(4-((6-Cyclohexyl-[1,2,4]triazolo[1,5-*a*]pyrazin-8-yl)amino)-1*H*-pyrazol-1-yl)cyclohexanol (1)

A flask equipped with a mechanical stirrer was charged with (1*r*,4*r*)-4-(4-((6-bromo-[1,2,4]triazolo[1,5-*a*]pyrazin-8-yl)amino)-1*H*-pyrazol-1-yl)cyclohexanol (83.7 g, 221 mmol), cesium carbonate (159 g, 487 mmol) and cyclohex-1-en-1-ylboronic acid (29.8 g, 237 mmol). 1,4-Dioxane (506 mL) and water (126 mL) were added. PdCl_2_(PPh_3_)_2_ (6.37 g, 9.07 mmol) was added and the reaction mixture was degassed and purged with nitrogen. The reaction mixture was heated to 90°C for 45 min and then cooled to ambient temperature overnight. To the reaction mixture was added water (1.4 L). The resultant suspension was stirred for 30 min, filtered, and washed with water (500 mL) and ethanol (1.2 L). The collected solid was dried under vacuum to give the title compound (84.1 g, 100%); MS *m/z*: 380 (M+H)^+^. ^1^H NMR (400 MHz, DMSO-*d*_6_) δ 10.12 (s, 1H), 8.49 (s, 1H), 8.21–8.09 (m, 2H), 7.82 (d, *J* = 0.6 Hz, 1H), 6.84 (q, *J* = 4.2, 3.5 Hz, 1H), 4.62 (d, *J* = 4.3 Hz, 1H), 4.11 (tt, *J* = 11.5, 3.9 Hz, 1H), 3.48 (tt, *J* = 10.8, 4.2 Hz, 1H), 2.41 (s, 2H), 2.26 (d, *J* = 5.8 Hz, 2H), 2.07–1.85 (m, 4H), 1.84–1.57 (m, 6H), 1.43–1.28 (m, 1H).

A 2 L stainless steel flask was charged with 20% Pd(OH)_2_/C (3.71 g, 5.29 mmol). A suspension of (1*r*,4*r*)-4-(4-((6-(cyclohex-1-en-1-yl)-[1,2,4]triazolo[1,5-*a*]pyrazin-8-yl)amino)-1*H*-pyrazol-1-yl)cyclohexanol (11.8 g, 31.1 mmol) in THF (244 mL), MeOH (122 mL) and acetic acid (61 mL) was then added under nitrogen. The flask was charged with 50 psi hydrogen on a Parr shaker at 50°C. Shaking was then continued for 12 h. The mixture was allowed to cool to ambient temperature and purged with nitrogen. The suspension was filtered through Celite® and washed with MeOH (1 L) and EtOAc (0.5 L). The filtrate was concentrated under vacuum to ~70 mL volume. Water was added until a thick precipitate formed. The suspension was filtered, washed with additional water and dried to give the title compound (10.1 g, 85%) as an off-white solid; ^1^H NMR (400 MHz, DMSO-*d*_6_) δ 10.14 (s, 1H), 8.47 (s, 1H), 8.19 (s, 1H), 8.04 (s, 1H), 7.83 (d, *J* = 0.6 Hz, 1H), 4.64 (d, *J* = 4.3 Hz, 1H), 4.10 (tt, *J* = 11.5, 3.9 Hz, 1H), 3.48 (ddt, *J* = 14.7, 10.6, 4.2 Hz, 1H), 2.61 (tt, *J* = 11.6, 3.5 Hz, 1H), 2.03 (d, *J* = 12.3 Hz, 2H), 1.96–1.50 (m, 11H), 1.49–1.15 (m, 5H); MS *m/z*: 382 (M+H)^+^.

#### 4-(4-((6-(Cyclohex-1-en-1-yl)-[1,2,4]triazolo[1,5-*a*]pyrazin-8-yl)amino)-1*H*-pyrazol-1-yl)butan-2-one (33)

But-3-en-2-one (0.721 mL, 8.89 mmol) was added to a solution of 6-(cyclohex-1-en-1-yl)-*N*-(1*H*-pyrazol-4-yl)-[1,2,4]triazolo[1,5-*a*]pyrazin-8-amine (2.50 g, 8.89 mmol) in DMF (60 mL). Cesium carbonate (2.90 g, 8.89 mmol) was then added and the reaction mixture was stirred at ambient temperature for 3 h. Additional but-3-en-2-one (0.020 mL, 0.243 mmol) was added and the reaction mixture was stirred an additional 1 h. Water (100 mL) and EtOAc (100 mL) were added and the organic layer was separated. The aqueous layer was extracted with EtOAc (100 mL). The combined organic layers were then dried over MgSO_4_, filtered, and concentrated to dryness. DCM (10 mL) was added and the suspension was filtered. The filtrate was concentrated. Purification by flash column chromatography (0–5% MeOH/DCM) gave the title compound (2.27 g, 73%) MS *m/z*: 352 (M+H)^+^; ^1^H NMR (400 MHz, DMSO-*d*_6_) δ 10.12 (s, 1H), 8.49 (s, 1H), 8.15 (s, 1H), 8.10–8.09 (m, 1H), 7.79–7.78 (m, 1H), 6.90–6.82 (m, 1H), 4.29 (t, *J* = 6.5 Hz, 2H), 2.99 (t, *J* = 6.5 Hz, 2H), 2.45–2.36 (m, 2H), 2.33–2.24 (m, 2H), 2.11 (s, 3H), 1.80–1.70 (m, 2H), 1.69–1.59 (m, 2H).

#### 4-(4-((6-Cyclohexyl-[1,2,4]triazolo[1,5-*a*]pyrazin-8-yl)amino)-1*H*-pyrazol-1-yl)-2-methylbutan-2-ol (5)

4-(4-((6-(Cyclohex-1-en-1-yl)-[1,2,4]triazolo[1,5-*a*]pyrazin-8-yl)amino)-1*H*-pyrazol-1-yl)butan-2-one (0.345 g, 0.982 mmol) was dissolved in THF (6 mL). Methylmagnesium bromide (3M solution in diethyl ether) (1.31 mL, 3.93 mmol) was added dropwise at ambient temperature and the mixture was stirred overnight. Additional methylmagnesium bromide (3 M solution in diethyl ether) (0.491 mL, 1.47 mmol) was added dropwise to this solution and the reaction mixture was stirred an additional 20 min. Saturated aqueous ammonium chloride (10 mL) was added and the mixture was extracted with EtOAc (2 x 25 mL). The combined organic layers were dried over MgSO_4_, filtered, and concentrated. The resultant residue was purified by flash column chromatography (100% EtOAc) to provide 4-(4-((6-(cyclohex-1-en-1-yl)-[1,2,4]triazolo[1,5-*a*]pyrazin-8-yl)amino)-1*H*-pyrazol-1-yl)-2-methylbutan-2-ol (0.136 g, 38%) MS *m/z*: 368 (M+H)^+^. ^1^H NMR (400 MHz, DMSO-*d*_6_) δ 10.12 (s, 1H), 8.49 (s, 1H), 8.16 (s, 1H), 8.11–8.09 (m, 1H), 7.80–7.79 (m, 1H), 6.88–6.80 (m, 1H), 4.41 (s, 1H), 4.19–4.13 (m, 2H), 2.44–2.37 (m, 2H), 2.29–2.22 (m, 2H), 1.95–1.85 (m, 2H), 1.80–1.70 (m, 2H), 1.68–1.58 (m, 2H), 1.12 (s, 6H).

To 4-(4-((6-(cyclohex-1-en-1-yl)-[1,2,4]triazolo[1,5-*a*]pyrazin-8-yl)amino)-1*H*-pyrazol-1-yl)-2-methylbutan-2-ol (1.21 g, 3.30 mmol) was added MeOH (20 mL), EtOAc (10 mL), and acetic acid (8 mL). 20% Pd(OH)_2_/C (0.810 g, 1.153 mmol) was added and the mixture stirred under H_2_ (1 atm) for 2 d. The mixture was filtered through Celite® and rinsed with MeOH and EtOAc. The filtrate was concentrated and the residue was purified by flash column chromatography (50–100% EtOAc/DCM) to provide the title compound (0.75 g, 62%) MS *m/z*: 370 (M+H)^+^; ^1^H NMR (400 MHz, DMSO-*d*_6_) δ 10.12 (s, 1H), 8.47 (s, 1H), 8.15–8.13 (m, 1H), 8.04–8.02 (m, 1H), 7.82–7.79 (m, 1H), 4.19–4.13 (m, 2H), 2.66–2.56 (m, 1H), 1.95–1.86 (m, 4H), 1.85–1.78 (m, 2H), 1.77–1.69 (m, 1H), 1.65–1.52 (m, 2H), 1.45–1.32 (m, 2H), 1.31–1.21 (m, 1H), 1.12 (s, 6H).

#### (1*r*,4*r*)-Ethyl 4-(4-nitro-1*H*-pyrazol-1-yl)cyclohexanecarboxylate (35)

(1*s*,4*s*)-Ethyl 4-hydroxycyclohexanecarboxylate (5.0 g, 29.0 mmol) was dissolved in pyridine (25 mL) and the resultant solution was cooled to 0°C. 4-Methylbenzene-1-sulfonyl chloride (6.09 g, 31.9 mmol) was added. The reaction mixture was allowed to warm to ambient temperature and stir for 17 h. The reaction mixture was added to water (150 mL) and extracted with EtOAc (100 mL). The organic layer was dried over MgSO_4_, filtered, and concentrated to give a colorless oil. The residue was purified by flash column chromatography (0–50% EtOAc/DCM) to provide (1*s*,4*s*)-ethyl 4-(tosyloxy)cyclohexanecarboxylate (7.6 g, 80%). ^1^H NMR (400 MHz, DMSO-*d*_6_) δ 7.78 (d, *J* = 8.3 Hz, 2H), 7.45 (d, *J* = 8.1 Hz, 2H), 4.68–4.60 (m, 1H), 4.03 (q, *J* = 7.1 Hz, 2H), 2.40 (s, 3H), 2.39–2.30 (m, 1H), 1.69–1.50 (m, 8H), 1.15 (t, *J* = 7.1 Hz, 3H).

(1*s*,4*s*)-Ethyl 4-(tosyloxy)cyclohexanecarboxylate (16.9 g, 51.8 mmol) was dissolved in DMF (174 mL). 4-Nitro-1*H*-pyrazole (3.90 g, 34.5 mmol) and cesium carbonate (12.4 g, 38.0 mmol) were added. The mixture was heated to 90°C for 5 h and then cooled to ambient temperature and stirred for 17 h. The mixture was poured into 250 mL of ice water, sonicated and filtered. The collected solids were rinsed with water (50 mL), then dried to provide the title compound (6.93 g, 75%) as a white solid. ^1^H NMR (400 MHz, DMSO-*d*_6_) δ 8.88 (s, 1H), 8.25 (s, 1H), 4.28 (tt, *J* = 11.9, 3.9 Hz, 1H), 4.07 (q, *J* = 7.1 Hz, 2H), 2.36 (tt, *J* = 12.1, 3.6 Hz, 1H), 2.13–2.00 (m, 4H), 1.88–1.75 (m, 2H), 1.52 (qd, *J* = 13.1, 3.3 Hz, 2H), 1.19 (t, *J* = 7.1 Hz, 3H).

#### (1*r*,4*r*)-Ethyl 4-(4-((6-bromo-[1,2,4]triazolo[1,5-*a*]pyrazin-8-yl)amino)-1*H*-pyrazol-1-yl)cyclohexanecarboxylate (36)

(1*r*,4*r*)-Ethyl 4-(4-nitro-1*H*-pyrazol-1-yl)cyclohexanecarboxylate (9.01 g, 33.7 mmol) was flushed with N_2_ in a flask. 10% Pd/C (1.50 g, 1.410 mmol) was added and the mixture was further flushed with N_2_. EtOH (150 mL) was added followed by THF (24 mL). The mixture was stirred under H2 (1 atm) for 17 h. The mixture was filtered through a pad of Celite® and rinsed with MeOH. The filtrate was concentrated to give (1*r*,4*r*)-ethyl 4-(4-amino-1*H*-pyrazol-1-yl)cyclohexanecarboxylate (7.41 g, 93%) as a pink solid. MS *m/z*: 238 (M+H)^+^. ^1^H NMR (400 MHz, DMSO-*d*_6_) δ 7.02 (s, 1H), 6.88 (s, 1H), 4.06 (q, *J* = 7.1 Hz, 2H), 3.93 (tt, *J* = 11.9, 3.5 Hz, 1H), 3.73 (br, 2H), 2.33 (tt, *J* = 12.3, 3.4 Hz, 1H), 2.03–1.91 (m, 4H), 1.75–1.61 (m, 2H), 1.55–1.40 (m, 2H), 1.18 (t, *J* = 7.1 Hz, 3H).

6,8-Dibromo-[1,2,4]triazolo[1,5-*a*]pyrazine (8.68 g, 31.2 mmol) was dissolved in DMF (84 mL). To this was added (1*r*,4*r*)-ethyl 4-(4-amino-1*H*-pyrazol-1-yl)cyclohexanecarboxylate (7.41 g, 31.2 mmol) and triethylamine (8.70 mL, 62.5 mmol). The reaction mixture was stirred at ambient temperature for 17 h. The reaction mixture was then added to ice water (200 mL) and extracted with EtOAc (200 mL). The organic layer was dried over MgSO_4_, filtered, and concentrated to provide the title compound (13.2 g, 97%). MS *m/z*: 436, 434 (M+H)^+^. ^1^H NMR (400 MHz, DMSO-*d*_6_) δ 10.66 (s, 1H), 8.56 (d, *J* = 6.2 Hz, 2H), 8.10 (s, 1H), 7.80 (s, 1H), 4.27–4.13 (m, 1H), 4.08 (q, *J* = 7.1 Hz, 2H), 2.40 (ddd, *J* = 12.3, 8.8, 3.4 Hz, 1H), 2.14–1.96 (m, 4H), 1.86–1.70 (m, 2H), 1.62–1.47 (m, 2H), 1.20 (t, *J* = 7.1 Hz, 3H).

#### (1*r*,4*r*)-Ethyl 4-(4-((6-cyclohexyl-[1,2,4]triazolo[1,5-*a*]pyrazin-8-yl)amino)-1*H*-pyrazol-1-yl)cyclohexanecarboxylate (37)

To a flask was added (1*r*,4*r*)-ethyl 4-(4-((6-bromo-[1,2,4]triazolo[1,5-*a*]pyrazin-8-yl)amino)-1*H*-pyrazol-1-yl)cyclohexanecarboxylate (13.2 g, 30.4 mmol), cyclohex-1-en-1-ylboronic acid (4.60 g, 36.5 mmol) and cesium carbonate (24.8 g, 76 mmol). 1,4–1,4-dioxane (80 mL) and water (20 mL) were added. The reaction mixture was evacuated and purged with N_2_. PdCl_2_(PPh_3_)_2_ (1.07 g, 1.52 mmol) was then added and mixture was heated to 90°C for 5 h and then stirred at ambient temperature for 17 h. Aqueous sodium bicarbonate solution (200 mL) was added. The resultant mixture was extracted with DCM (2 x 200 mL). The combined organic layers were dried over MgSO_4_, filtered, and concentrated. To the resultant residue was added DCM (15 mL). The suspension was filtered and rinsed with DCM. The collected solids were dried to provide 7.51 g of product. The filtrate was concentrated, then purified via flash column chromatography (0–3% MeOH/DCM) to give additional product. The two lots of product were combined to give (1*r*,4*r*)-ethyl 4-(4-((6-bromo-[1,2,4]triazolo[1,5-*a*]pyrazin-8-yl)amino)-1*H*-pyrazol-1-yl)cyclohexanecarboxylate (11.8 g, 89%). MS *m/z*: 436 (M+H)^+^. ^1^H NMR (400 MHz, DMSO-*d*_6_) δ 10.13 (s, 1H), 8.14 (d, *J* = 14.2 Hz, 2H), 7.84 (s, 1H), 6.84 (s, 1H), 4.15 (tt, *J* = 11.6, 3.8 Hz, 1H), 4.11–4.01 (m, 2H), 2.44–2.32 (m, 3H), 2.32–2.21 (m, 2H), 2.10 (dd, *J* = 13.1, 3.6 Hz, 2H), 2.02 (dd, *J* = 14.0, 3.5 Hz, 2H), 1.83–1.59 (m, 7H), 1.60–1.47 (m, 2H), 1.18 (t, *J* = 7.1 Hz, 3H).

To (1*r*,4*r*)-ethyl 4-(4-((6-(cyclohex-1-en-1-yl)-[1,2,4]triazolo[1,5-*a*]pyrazin-8-yl)amino)-1*H*-pyrazol-1-yl)cyclohexanecarboxylate (11.8 g, 27.2 mmol) was added MeOH (136 mL) and THF (136 mL). 20% Pd(OH)_2_/C (2.86 g, 4.07 mmol) was added. The flask was charged with hydrogen (55 psi) on a Parr shaker at 45°C. Shaking was then continued for 18 h. The mixture was allowed to cool to ambient temperature and purged with nitrogen. The reaction mixture was filtered through Celite® and flushed with methanol and THF. The filtrate was concentrated to provide the title compound (10.4 g, 87%). MS *m/z*: 438 (M+H)^+^; ^1^H NMR (400 MHz, DMSO-*d*_6_) δ 10.13 (s, 1H), 8.17 (s, 1H), 8.04 (s, 1H), 7.86 (s, 1H), 4.14 (tt, *J* = 11.6, 3.8 Hz, 1H), 4.06 (t, *J* = 7.1 Hz, 2H), 3.27 (s, 4H), 2.66–2.55 (m, 1H), 2.37 (ddd, *J* = 12.0, 8.4, 3.6 Hz, 1H), 2.15–2.07 (m, 2H), 2.06–1.98 (m, 2H), 1.95–1.87 (m, 2H), 1.86–1.78 (m, 2H), 1.77–1.69 (m, 2H), 1.65–1.50 (m, 4H), 1.45–1.33 (m, 2H), 1.32–1.21 (m, 1H), 1.18 (t, *J* = 7.1 Hz, 3H).

#### ((1*r*,4*r*)-4-(4-((6-Cyclohexyl-[1,2,4]triazolo[1,5-*a*]pyrazin-8-yl)amino)-1*H*-pyrazol-1-yl)cyclohexanecarboxylic acid (6)

To (1*r*,4*r*)-ethyl 4-(4-((6-cyclohexyl-[1,2,4]triazolo[1,5-*a*]pyrazin-8-yl)amino)-1*H*-pyrazol-1-yl)cyclohexanecarboxylate (10.38 g, 23.72 mmol) was added MeOH (65 mL). NaOH (2M in H_2_O 35.6 mL, 71.2 mmol) was added and the mixture was stirred for 17 h. 1N HCl was added until a precipitate formed. The resultant suspension was filtered. The collected solid was rinsed with water and dried to provide the title compound (9.36 g, 96%) as a white solid. MS *m/z*: 410 (M+H)^+^; ^1^H NMR (400 MHz, DMSO-*d*_6_) δ 12.09 (s, 1H), 10.13 (s, 1H), 8.17 (s, 1H), 8.04 (s, 1H), 7.85 (s, 1H), 4.13 (tt, *J* = 11.6, 3.9 Hz, 1H), 2.61 (tt, *J* = 11.6, 3.5 Hz, 1H), 2.27 (tt, *J* = 12.0, 3.6 Hz, 1H), 2.15–2.06 (m, 2H), 2.07–1.96 (m, 2H), 1.96–1.87 (m, 2H), 1.88–1.78 (m, 2H), 1.79–1.70 (m, 3H), 1.67–1.47 (m, 4H), 1.46–1.32 (m, 2H), 1.32–1.18 (m, 1H).

#### (1*r*,4*r*)-4-(4-((6-Cyclohexyl-[1,2,4]triazolo[1,5-*a*]pyrazin-8-yl)amino)-1*H*-pyrazol-1-yl)cyclohexyl 2-(dimethylamino)acetate (7)

(1*r*,4*r*)-4-(4-((6-Cyclohexyl-[1,2,4]triazolo[1,5-*a*]pyrazin-8-yl)amino)-1*H*-pyrazol-1-yl)cyclohexanol (0.10 g, 0.26 mmol), 2-(dimethylamino)acetic acid (0.054 g, 0.52 mmol), DMAP (0.0030 g, 0.026 mmol), EDC (0.101 g, 0.524 mmol) and DCM (5 mL) were combined to give a white suspension. The mixture was stirred at ambient temperature overnight. The resulting solution was washed with a saturated solution of NaHCO_3_ (2x5 mL). The organic layer was dried over MgSO_4_, filtered and concentrated under reduced pressure. The crude material was purified by flash column chromatography (0–10% MeOH/DCM) to afford the title compound (0.12 g, 92%) as white solid: MS *m/z* 467 (M+H)^+^; ^1^H NMR (DMSO-*d6*) δ 10.13 (s, 1H), 8.47 (s, 1H), 8.18 (s, 1H), 8.04 (s, 1H), 7.87 (s, 1H), 4.75 (m, 1H), 4.21 (m, 1H), 3.27 (s, 1H), 3.13 (s, 2H), 2.61 (m, 1H), 2.23 (s, 6H), 2.10 (d, *J* = 10.86 Hz, 2H), 2.01 (d, *J* = 9.65 Hz, 2H), 1.95–1.78 (m, 6H), 1.74 (m, 1H), 1.66–1.51 (m, 4H), 1.47–1.31 (m, 2H), 1.25 (m, 1H).

#### (*R*)-(1*r*,4*r*)-4-(4-((6-Cyclohexyl-[1,2,4]triazolo[1,5-*a*]pyrazin-8-yl)amino)-1*H*-pyrazol-1-yl)cyclohexyl 2-amino-3-methylbutanoate (8)

(1*r*,4*r*)-4-(4-((6-Cyclohexyl-[1,2,4]triazolo[1,5-*a*]pyrazin-8-yl)amino)-1*H*-pyrazol-1-yl)cyclohexanol (0.10 g, 0.262 mmol), (*R*)-2-((*tert*-butoxycarbonyl)amino)-3-methylbutanoic acid (0.085 g, 0.393 mmol), DMAP (3.2 mg, 0.026 mmol) and EDC (0.101 g, 0.524 mmol), and DCM (5.24 mL) were combined. The mixture was stirred at ambient temperature for 16 h. The reaction mixture was washed with saturated aqueous NaHCO_3_ (10 mL), dried over Na_2_SO_4_, filtered and concentrated. The residue was purified via flash column chromatography (0–10% MeOH/CH_2_Cl_2_) to afford (*R*)-(1*r*,4*r*)-4-(4-((6-cyclohexyl-[1,2,4]triazolo[1,5-*a*]pyrazin-8-yl)amino)-1*H*-pyrazol-1-yl)cyclohexyl 2-((*tert*-butoxycarbonyl)amino)-3-methylbutanoate (0.11 g, 72%) as a colorless wax.

(*R*)-(1*r*,4*r*)-4-(4-((6-Cyclohexyl-[1,2,4]triazolo[1,5-*a*]pyrazin-8-yl)amino)-1*H*-pyrazol-1-yl)cyclohexyl 2-((*tert*-butoxycarbonyl)amino)-3-methylbutanoate (0.11 g, 0.189 mmol) was dissolved in DCM (7.6 mL). The solution was cooled to 0°C. TFA (0.846 mL, 11.0 mmol) was added dropwise. The mixture was allowed to warm to ambient temperature and stirred for 16 h. The solution was concentrated. The residue was dissolved in EtOAc (10 mL), washed with saturated aqueous NaHCO_3_ (3x10 mL), brine (10 mL), dried over Na_2_SO_4_, filtered and concentrated to afford the title compound (0.068 g, 73%) as white solid. MS *m/z*: 481 (M+H)^+^. ^1^H NMR (400 MHz, DMSO-*d*_6_) δ 10.14 (s, 1H), 8.47 (s, 1H), 8.19 (d, *J* = 0.50 Hz, 1H), 8.06–8.01 (m, 1H), 7.86 (d, *J* = 0.60 Hz, 1H), 4.74 (ddd, *J* = 4.23, 10.73, 14.87 Hz, 1H), 4.21 (tt, *J* = 3.74, 10.96 Hz, 1H), 3.09 (d, *J* = 5.31 Hz, 1H), 2.61 (tt, *J* = 3.32, 11.66 Hz, 1H), 2.11 (d, *J* = 10.61 Hz, 2H), 2.00 (d, *J* = 10.37 Hz, 2H), 1.95–1.66 (m, 8H), 1.65–1.49 (m, 4H), 1.39 (qt, *J* = 3.15, 12.68 Hz, 2H), 1.32–1.19 (m, 1H), 0.85 (dd, *J* = 6.82, 18.78 Hz, 6H).

#### (2R,3R,4S,5S,6R)-2-(((1*r*,4*r*)-4-(4-((6-Cyclohexyl-[1,2,4]triazolo[1,5-a]pyrazin-8-yl)amino)-1*H*-pyrazol-1-yl)cyclohexyl)oxy)-6-(hydroxymethyl)tetrahydro-2*H*-pyran-3,4,5-triol and (2S,3R,4S,5S,6R)-2-(((1*r*,4*s*)-4-(4-((6-cyclohexyl-[1,2,4]triazolo[1,5-*a*]pyrazin-8-yl)amino)-1*H*-pyrazol-1-yl)cyclohexyl)oxy)-6-(hydroxymethyl)tetrahydro-2*H*-pyran-3,4,5-triol (9, 10)

A solution of (1*r*,4*r*)-4-(4-((6-cyclohexyl-[1,2,4]triazolo[1,5-*a*]pyrazin-8-yl)amino)-1*H*-pyrazol-1-yl)cyclohexanol (0.150 g, 0.393 mmol) and 2,3,4,6-tetra-*O*-benzyl-alpha-D-glucopyranosyl trichloroacetimidate (0.323 g, 0.472 mmol) in DCM (8 mL) was cooled to -78°C and (diethyloxonio)trifluoroborate (0.0097 mL, 0.079 mmol) was added. The reaction mixture was allowed to warm to ambient temperature and stirred overnight. The reaction mixture was recooled to -78°C. Additional (2*R*,3*R*,4*S*,5*R*,6*R*)-3,4,5-tris(benzyloxy)-6-((benzyloxy)methyl)tetrahydro-2*H*-pyran-2-yl 2,2,2-trichloroacetimidate (0.269 g, 0.393 mmol) and (diethyloxonio)trifluoroborate (0.020 mL, 0.157 mmol) were added. The mixture was allowed to warm to ambient temperature and stir for 8 h. The reaction mixture was concentrated. The residue was purified via flash column chromatography (0–3% MeOH/DCM) to give 6-cyclohexyl-*N*-(1-((1*r*,4*r*)-4-(((2*R*,3*R*,4*S*,5*R*,6*R*)-3,4,5-tris(benzyloxy)-6-((benzyloxy)methyl)tetrahydro-2*H*-pyran-2-yl)oxy)cyclohexyl)-1*H*-pyrazol-4-yl)-[1,2,4]triazolo[1,5-*a*]pyrazin-8-amine (0.336 g, 95%) as an off-white solid: MS *m/z* 904 (M+H)^+^; ^1^H NMR (DMSO-*d*_6_) δ 10.1 9s, 1 H), 8.47 (s, 1 H), 8.18 (s, 1 H), 8.06 (s, 1 H), 7.86 (s, 1 H), 7.37–7.25 (m, 18 H), 7.17 (d, *J* = 8 Hz, 2 H), 4.88 (m, 1 H), 4.81 (m, 1 H), 4.71 (m, 2 H), 4.65 (m,2 H), 4.53–4.46 (m, 3 H), 4.19 (m, 1 H), 3.76 (m, 1H), 3.68 (m, 1 H), 3.62–3.53 (m, 4 H), 3.43 (m, 2 H), 2.61 (m, 2 H), 2.16 (m, 4 H), 1.91 (m, 2 H), 1.84–1.69 (m, 6 H), 1.64–1.51 (m, 4 H), 1.43–1.32 (m, 3 H), 1.29–1.20 (m, 2 H).

A solution of 6-cyclohexyl-*N*-(1-((1*r*,4*r*)-4-(((2*R*,3*R*,4*S*,5*R*,6*R*)-3,4,5-tris(benzyloxy)-6-((benzyloxy)methyl)tetrahydro-2*H*-pyran-2-yl)oxy)cyclohexyl)-1*H*-pyrazol-4-yl)-[1,2,4]triazolo[1,5-*a*]pyrazin-8-amine (0.336 g, 0.372 mmol) in DCM (5 mL) and MeOH (5 mL) was passed through an H-cube (Pd/C, 10 bar H_2_, ambient temperature). The reaction mixture was concentrated and the residue was purified via chiral chromatography (Waters Chiral Prep, Daicel ID 20x250 mm column, 0.20% diethylamine in heptane/ethyl acetate) to give the title compound **9** (0.060 g, 30%): ^1^H NMR (DMSO-*d*_6_) δ 10.1 (br s, 1 H), 8.47 (s, 1 H), 8.17 (s, 1 H), 8.04 (s, 1 H), 7.86 (s, 1 H), 4.25 (d, *J* = 7.6 Hz, 1 H), 4.14 (m, 1 H), 3.69 (m, 1 H), 3.66 (dd, *J* = 11.8 Hz, *J* = 2.4 Hz, 1 H), 3.42 (dd, *J* = 11.6 Hz, *J* = 9.6 Hz, 1 H), 3.15–3.03 (m, 2 H), 3.03–3.01 (m, 2 H), 2.91 (m, 2 H), 2.61 (m, 2 H), 2.08 (m, 4 H), 1.91 (m, 2 H), 1.84–1.71 (m, 4 H), 1.63–1.52 (m, 3 H), 1.48–1.33 (m, 4 H), 1.28–1.21 (m, 2 H) and the title compound **10** (0.034 g, 16%): ^1^H NMR (DMSO-*d*_6_) δ 10.1 (s, 1 H), 8.47 (s, 1 H), 8.18 (s, 1 H), 8.04 (s, 1 H), 7.86 (s,1 H), 4.79 (m, 2 H), 4.65 (d, *J* = 4.8 Hz, 1 H), 4.46 (d, *J* = 5.2 Hz, 1 H), 4.36 (m, 1 H), 4.16 (m, 1 H), 3.60 (m, 1 H), 3.45 (m, 2 H), 3.39 (m, 1 H), 3.16 (m, 1 H), 3.04 (m, 1 H), 2.61 (m, 2 H), 2.07 (m, 4 H), 1.91 (m, 2 H), 1.85–1.72, m, 4 H), 1.63–1.50 (m, 3 H), 1.47–1.34 (m, 3 H), 1.25 (m, 2 H).

#### (2S,3S,4S,5R,6R)-6-(((1*r*,4*r*)-4-(4-((6-Cyclohexyl-[1,2,4]triazolo[1,5-*a*]pyrazin-8-yl)amino)-1*H*-pyrazol-1-yl)cyclohexyl)oxy)-3,4,5-trihydroxytetrahydro-2*H*-pyran-2-carboxylic acid (11)

A round bottom flask was charged with (1*r*,4*r*)-4-(4-((6-cyclohexyl-[1,2,4]triazolo[1,5-*a*]pyrazin-8-yl)amino)-1*H*-pyrazol-1-yl)cyclohexanol (0.30 g, 0.79 mmol) and DCM (4 mL) to give an off-white suspension. The reaction mixture was cooled to 5°C. (2*R*,3*R*,4S,5*S*,6*S*)-2-bromo-6-(methoxycarbonyl)tetrahydro-2*H*-pyran-3,4,5-triyl triacetate (0.38 g, 0.94 mmol) and silver(I) trifluoromethanesulfonate (0.20 g, 0.79 mmol) were added. The cooling bath was removed and the reaction was stirred at ambient temperature overnight. The reaction was then diluted with the DCM and water. The organic layer was separated, dried over MgSO_4_, filtered and concentrated under reduced pressure. The crude material was purified by flash column chromatography (0–100% EtOAc/DCM). The resultant material was then purified by HPLC-MS to give (3*R*,4*S*,5*S*,6*S*)-2-(((1*r*,4*r*)-4-(4-((6-cyclohexyl-[1,2,4]triazolo[1,5-*a*]pyrazin-8-yl)amino)-1*H*-pyrazol-1-yl)cyclohexyl)oxy)-6-(methoxycarbonyl)tetrahydro-2*H*-pyran-3,4,5-triyl triacetate (0.04 g, 7%) as a pale yellow solid: MS *m/z* 698 (M+H)^+^; ^1^H NMR (DMSO-*d*_6_) δ 10.1 (s, 1 H), 8.47 (s, 1 H), 8.16 (s,1 H), 8.04 (s, 1 H), 7.85 (s, 1 H), 5.32 (t, 1 H, *J* = 9.6 Hz), 5.02 (d, 1 H, *J* = 8.0 Hz), 4.94 (t, 1 H, *J* = 9.6 Hz), 4.75 (dd, 1 H, *J* = 8 Hz, *J* = 7.6 Hz), 4.45 (d, 1 H, *J* = 9.6 Hz), 4.15 (m, 1 H), 3.67 (m, 1 H), 3.63 (s, 3 H), 2.61 (m, 1 H), 2.09–2.01 (m, 3 H), 2.01 (s, 3 H), 1.97 (s, 3 H), 1.95 (s, 3 H), 1.91 (m, 2 H), 1.86–1.78 (m, 3 H), 1.77–1.71 (m, 2 H), 1.64–1.51 (m, 2 H), 1.48–1.33 (m, 4 H), 1.25 (m, 2 H).

A round bottom flask was charged with (3*R*,4*S*,5*S*,6*S*)-2-(((1*r*,4*r*)-4-(4-((6-cyclohexyl-[1,2,4]triazolo[1,5-*a*]pyrazin-8-yl)amino)-1*H*-pyrazol-1-yl)cyclohexyl)oxy)-6-(methoxycarbonyl)tetrahydro-2*H*-pyran-3,4,5-triyl triacetate (0.040 g, 0.057 mmol), THF (2 mL) and water (1 mL). The resultant suspension was cooled to 0°C. Lithium hydroxide monohydrate (0.012 g, 0.29 mmol) was added and the reaction mixture was stirred at 0–10°C for 1 h. Water (20 mL) was added and the mixture was extracted with a solution of 20% IPA/DCM (3x50 mL). The aqueous and organic layers were combined and concentrated. The resultant residue was purified via HPLC-MS to give the title compound (0.017 g, 50%) as an off-white solid: MS *m/z* 558 (M+H)^+^; ^1^H NMR (DMSO-*d*_6_) δ 10.1 (s, 1 H), 8.47 (s, 1 H), 8.17 (s, 1 H), 8.04 (s, 1 H), 7.86 (s, 1 H), 4.95 (d, 1 H, *J* = 8 Hz), 4.33 (d, 1 H, *J* = 7.6 Hz), 4.13 (m, 2 H), 4.01 (dd, 1 H, *J* = 7.2 Hz, *J* = 7.2 Hz), 3.68–3.61 (m, 2 H), 3.55 (m, 1 H), 3.18–3.12 (m, 1 H), 3.15 (d, 1 H), *J* = 5.2 Hz), 2.95 (m, 1 H), 2.08 (m, 3 H), 1.91 (m, 2 H), 1.85–1.71 (m, 5 H), 1.65–1.49 (m, 3 H), 1.46–1.34 (m, 4 H), 1.26 (m 2 H).

#### α-cyclodextrin conjugate 21

(1*r*,4*r*)-Ethyl 4-(4-((6-cyclohexyl-[1,2,4]triazolo[1,5-*a*]pyrazin-8-yl)amino)-1*H*-pyrazol-1-yl)cyclohexanecarboxylate (0.219 g, 0.5 mmol) was suspended in acetonitrile (10 mL) and water (2 mL). NaOH (2.0 M in water, 0.250 mL, 0.500 mmol) was added and the mixture was stirred at room temperature overnight. The mixture was lyophilized. The resultant solid was suspended in DMA (2.5 mL). Mono-6-*O*-(*p*-toluenesulfonyl)-α-cyclodextrin (0.564 g, 0.500 mmol) was added. The mixture was heated at 100°C overnight. The mixture was cooled to ambient temperature and directly purified via HPLC-MS to give α-cyclodextrin conjugate **21** (0.24 g, 33%) as a light brown solid. MS *m/z*: 1365 (M+H)^+^. ^1^H NMR (400 MHz, DMSO-*d*_6_) δ 10.12 (s, 1H), 8.47 (s, 1H), 8.14 (s, 1H), 8.04 (s, 1H), 7.89 (s, 1H), 5.62–5.34 (m, 12H), 4.89–4.71 (m, 6H), 4.58–4.39 (m, 4H), 4.37–4.26 (m, 2H), 4.24–4.41 (m, 2H), 3.93–3.87 (m, 1H), 3.83–3.73 (m, 6H), 3.72–3.51 (m, 15H), 3.46–3.29 (m, 12H), 2.69–2.55 (m, 1H), 2.47–2.38 (m, 1H), 2.17–1.97 (m, 4H), 1.91 (d, *J* = 11.9 Hz, 2H), 1.87–1.67 (m, 5H), 1.67–1.48 (m, 4H), 1.39 (q, *J* = 12.7 Hz, 2H), 1.32–1.17 (m, 1H).

#### β-cyclodextrin conjugate 22

(1*r*,4*r*)-Ethyl 4-(4-((6-cyclohexyl-[1,2,4]triazolo[1,5-*a*]pyrazin-8-yl)amino)-1*H*-pyrazol-1-yl)cyclohexanecarboxylate (0.219 g, 0.501 mmol) was suspended in acetonitrile (10 mL) and water (2 mL). NaOH (2.0 M in water, 0.250 mL, 0.501 mmol) was added and the mixture was stirred at room temperature overnight. The reaction mixture was lyophilized. The residue was dissolved in DMA (2.5 mL). Mono-6-*O*-(*p*-toluenesulfonyl)-β-cyclodextrin (0.80 g, 0.621 mmol) was added. The mixture was heated to 100°C overnight. The mixture was cooled to ambient temperature and filtered through a 0.4 micron syringe filter. The resultant solution was directly purified via HPLC-MS to give β cyclodextrin conjugate **22** (182 mg, 23%) as a white solid. MS *m/z*: 1527 (M+H)^+^. ^1^H NMR (400 MHz, DMSO-*d*_6_) δ 10.13 (s, 1H), 8.47 (s, 1H), 8.13 (s, 1H), 8.04 (s, 1H), 7.89 (s, 1H), 5.85–5.61 (m, 14H), 4.90–4.76 (m, 7H), 4.48 (t, *J* = 5.8 Hz, 1H), 4.45–4.38 (m, 4H), 4.33–4.24 (m, 2H), 4.20–4.09 (m, 2H), 3.90–3.83 (m, 1H), 3.71–3.49 (m, 25H), 3.43–3.30 (m, 14H), 2.67–2.55 (m, 1H), 2.47–2.38 (m, 1H), 2.15–1.97 (m, 4H), 1.91 (d, *J* = 11.3 Hz, 2H), 1.82 (d, *J* = 12.8 Hz, 2H), 1.78–1.69 (m, 3H), 1.65–1.49 (m, 4H), 1.39 (q, *J* = 12.5 Hz, 2H), 1.26 (t, *J* = 12.4 Hz, 1H).

#### γ-cyclodextrin conjugate 23

(1*r*,4*r*)-4-(4-((6-Cyclohexyl-[1,2,4]triazolo[1,5-*a*]pyrazin-8-yl)amino)-1*H*-pyrazol-1-yl)cyclohexanecarboxylic acid (618 mg, 1.21 mmol) was suspended in acetonitrile (32 mL). NaOH (2.0 M in water, 0.724 mL, 1.45 mmol) was added. The resultant suspension was lyophilized. To the resultant residue was added mono-6-*O*-mesitylenesulfonyl-γ-cyclodextrin (3.57 g, 2.42 mmol). DMA (8 mL) was added. The reaction mixture was heated to 100°C overnight. The reaction mixture was cooled to ambient temperature and purified via HPLC-MS to give the γ-cyclodextrin conjugate **23** (0.588 g, 27%) as a white solid. MS *m/z*: 1689 (M+H)^+^. ^1^H NMR (400 MHz, DMSO-*d*_6_) δ 10.12 (s, 1H), 8.47 (s, 1H), 8.14 (s, 1H), 8.04 (s, 1H), 7.89 (s, 1H), 5.90–5.63 (m, 16H), 4.95–4.82 (m, 8H), 4.59–4.39 (m, 6H), 4.38–4.24 (m, 2H), 4.20–4.07 (m, 2H), 3.86–3.77 (m, 1H), 3.74–3.46 (m, 29H), 3.46–3.30 (m, 16H), 2.61 (t, *J* = 11.9 Hz, 1H), 2.42 (d, *J* = 11.9 Hz, 1H), 2.16–1.97 (m, 4H), 1.91 (d, *J* = 11.4 Hz, 2H), 1.83 (d, *J* = 12.7 Hz, 2H), 1.78–1.68 (m, 3H), 1.68–1.48 (m, 4H), 1.39 (q, *J* = 12.7 Hz, 2H), 1.31–1.20 (m, 1H). Starting material was also recovered from the HPLC-MS purification; obtained (1*r*,4*r*)-4-(4-((6-cyclohexyl-[1,2,4]triazolo[1,5-*a*]pyrazin-8-yl)amino)-1*H*-pyrazol-1-yl)cyclohexanecarboxylic acid (0.115 g, 23%) as a white solid.

#### 4-(((1*r*,4*r*)-4-(4-((6-Cyclohexyl-[1,2,4]triazolo[1,5-*a*]pyrazin-8-yl)amino)-1*H*-pyrazol-1-yl)cyclohexyl)oxy)-4-oxobutanoic acid (15)

A 500 mL flask was charged with (1*r*,4*r*)-4-(4-((6-cyclohexyl-[1,2,4]triazolo[1,5-*a*]pyrazin-8-yl)amino)-1*H*-pyrazol-1-yl)cyclohexanol (10.1 g, 26.5 mmol), DCM (265 mL) and triethylamine (11.1 mL, 79 mmol). Succinic anhydride (3.97 g, 39.7 mmol) and DMAP (0.323 g, 2.65 mmol) were then added. The reaction mixture was stirred at ambient temperature overnight. Additional succinic anhydride (0.530 g, 5.30 mmol) was added and the reaction mixture was stirred for an additional 12 h. The reaction mixture was diluted with 1N HCl (200 mL) and the resulting suspension was filtered. The collected solid was washed with 10% MeOH/DCM (50 mL) and water (50 mL). The solid was then dried under vacuum to give the title compound (12.5 g, 98%) as a white solid; MS *m/z*: 482 (M+H)^+^. ^1^H NMR (400 MHz, DMSO-*d*_6_) δ 12.18 (s, 1H), 10.15 (s, 1H), 8.47 (s, 1H), 8.19 (s, 1H), 8.04 (s, 1H), 7.86 (s, 1H), 4.71 (td, *J* = 10.5, 5.2 Hz, 1H), 4.20 (ddt, *J* = 11.0, 7.6, 4.1 Hz, 1H), 2.68–2.55 (m, 1H), 2.18–1.10 (m, 19H).

#### α-cyclodextrin conjugate 2

A 1 L flask was charged with 4-(((1*r*,4*r*)-4-(4-((6-cyclohexyl-[1,2,4]triazolo[1,5-*a*]pyrazin-8-yl)amino)-1*H*-pyrazol-1-yl)cyclohexyl)oxy)-4-oxobutanoic acid (11.5 g, 23.9 mmol), α-cyclodextrin (46.5 g, 47.8 mmol) and DMAP (0.584 g, 4.78 mmol). DMF (239 mL) and EDC (6.87 g, 35.8 mmol) were added and the reaction mixture was stirred at ambient temperature for 5 h. To the reaction mixture was added acetone (1 L). The resultant suspension was allowed to settle overnight. The reaction mixture was then filtered and rinsed with ether (200 mL). The precipitate was dried in a vacuum oven at 60°C for 3 h. The crude material was purified via HPLC-MS and lyophilized to give α-cyclodextrin conjugate **2** (11.1 g, 32%) as a white solid; MS *m/z*: 1436 (M+H)^+^. ^1^H NMR (400 MHz, DMSO-*d*_6_) δ 10.13 (s, 1H), 8.47 (s, 1H), 8.17 (s, 1H), 8.04 (s, 1H), 7.87 (s, 1H), 5.60–5.32 (m, 12H), 4.88–4.75 (m, 6H), 4.70 (dt, *J* = 10.6, 6.3 Hz, 1H), 4.59–4.37 (m, 5H), 4.35–4.15 (m, 3H), 3.83–3.72 (m, 6H), 3.72–3.51 (m, 18 H), 3.44–3.30 (m, 10H), 2.67–2.51 (m, 5H), 2.10 (d, *J* = 9.9 Hz, 2H), 2.00 (d, *J* = 9.3 Hz, 2H), 1.91 (d, *J* = 13.3 Hz, 2H), 1.87–1.78 (m, 4H), 1.74 (d, *J* = 12.0 Hz, 1H), 1.66–1.49 (m, 4H), 1.39 (q, *J* = 12.8 Hz, 2H), 1.25 (t, *J* = 12.6 Hz, 1H).

#### β-cyclodextrin conjugate 12

4-(((1*r*,4*r*)-4-(4-((6-Cyclohexyl-[1,2,4]triazolo[1,5-*a*]pyrazin-8-yl)amino)-1*H*-pyrazol-1-yl)cyclohexyl)oxy)-4-oxobutanoic acid (0.240 g, 0.498 mmol), β-cyclodextrin (1.13 g, 0.997 mmol), EDC (0.115 g, 0.598 mmol) and DMAP (12.2 mg, 0.100 mmol) were combined. DMF (5 mL) was added and the reaction mixture was stirred at ambient temperature for 18 h. Acetone (15 mL) was added and the resultant suspension was filtered, rinsing with acetone (2 x 10 mL). The collected solid was purified via HPLC-MS to obtain β- cyclodextrin conjugate **12** (0.101 g, 11%) as a white solid. MS *m/z*: 1599 (M+H)^+^. ^1^H NMR (400 MHz, DMSO-*d*_6_) δ 10.13 (s, 1H), 8.47 (d, *J* = 2.2 Hz, 1H), 8.17 (d, *J* = 5.3 Hz, 1H), 8.04 (s, 1H), 7.87 (d, *J* = 3.5 Hz, 1H), 5.84–5.59 (m, 14H), 4.83 (d, *J* = 13.3 Hz, 7H), 4.77–4.65 (m, 1H), 4.65–4.13 (m, 10H), 3.93–3.80 (m, 2H), 3.75–3.45 (m, 25H), 3.45–3.30 (m, 13H), 2.78–2.51 (m, 5H), 2.10 (d, *J* = 12.5 Hz, 2H), 2.00 (d, *J* = 8.6 Hz, 2H), 1.91 (d, *J* = 13.7 Hz, 2H), 1.88–1.78 (m, 3H), 1.74 (d, *J* = 12.6 Hz, 1H), 1.59 (d, *J* = 9.2 Hz, 3H), 1.51–1.32 (m, 2H), 1.32–1.15 (m, 2H).

#### γ-cyclodextrin conjugate 13

4-(((1*r*,4*r*)-4-(4-((6-Cyclohexyl-[1,2,4]triazolo[1,5-*a*]pyrazin-8-yl)amino)-1*H*-pyrazol-1-yl)cyclohexyl)oxy)-4-oxobutanoic acid (250 mg, 0.519 mmol), EDC (139 mg, 0.727 mmol), γ-cyclodextrin (1.35 g, 1.04 mmol) and DMAP (12.7 mg, 0.104 mmol) were combined. DMF (5.2 mL) was added and the reaction mixture was stirred at ambient temperature for 4 h. Acetone (15 mL) was added. The resultant suspension was filtered, rinsing with acetone (2 x 10 mL). The collected solid was purified via HPLC-MS obtain γ-cyclodextrin conjugate **13** (0.195 g, 21%) as a white solid. MS *m/z*: 1762 (M+H)^+^. ^1^H NMR (400 MHz, DMSO-*d*_6_) δ 10.13 (s, 1H), 8.47 (s, 1H), 8.17 (s, 1H), 8.04 (s, 1H), 7.88 (s, 1H), 5.98–5.59 (m, 16H), 4.87 (s, 8H), 4.79–4.64 (m, 1H), 4.47 (s, 8H), 4.30 (d, *J* = 10.9 Hz, 2H), 4.27–4.07 (m, 2H), 3.79 (s, 2H), 3.75–3.45 (m, 27H), 3.45–3.22 (m, 17H), 2.73–2.51 (m, 5H), 2.10 (d, *J* = 9.9 Hz, 2H), 2.06–1.95 (m, 2H), 1.91 (d, *J* = 12.8 Hz, 2H), 1.87–1.78 (m, 3H), 1.74 (d, *J* = 11.8 Hz, 1H), 1.67–1.51 (m, 3H), 1.39 (q, *J* = 12.6 Hz, 2H), 1.31–1.17 (m, 1H).

#### Dextran conjugate 14

4-(((1*r*,4*r*)-4-(4-((6-Cyclohexyl-[1,2,4]triazolo[1,5-*a*]pyrazin-8-yl)amino)-1*H*-pyrazol-1-yl)cyclohexyl)oxy)-4-oxobutanoic acid (0.300 g, 0.623 mmol) and CDI (0.177 g, 1.09 mmol) were dissolved in DMSO (1.56 mL). The reaction mixture was stirred for 90 min. ~70000 MW Dextran from *Leuconostoc* spp (5 wt% in DMSO, 18.6 mL, 0.623 mmol) and triethylamine (1.86 mL, 13.3 mmol) were added. The mixture was stirred at ambient temperature for 16 h. The mixture was diluted with 1:1 ethanol:ether (75 mL). The resultant suspension was filtered. The resultant tacky solid was immediately suspended in methanol (100 mL) and sonicated for 10 min. The resultant suspension was filtered and the collected solid was dried to give dextran conjugate **14** (0.710 g) as a white solid. A 10 mg/mL solution of the product in 9:9:2 DMSO:MeOH:2N aqueous NaOH was prepared and stirred for 3 h. LCMS quantitation of the resultant solution against a standard curve indicated a concentration of 1.15 mg/mL compound **1**.

#### 4-(2-(4-((6-Cyclohexyl-[1,2,4]triazolo[1,5-*a*]pyrazin-8-yl)amino)-1*H*-pyrazol-1-yl)ethoxy)-4-oxobutanoic acid (39)

To a suspension of 2-(4-((6-cyclohexyl-[1,2,4]triazolo[1,5-*a*]pyrazin-8-yl)amino)-1*H*-pyrazol-1-yl)ethanol (497 mg, 1.52 mmol) in DCM (9.0 mL), triethylamine (0.635 mL, 4.55 mmol) was added followed by succinic anhydride (190 mg, 1.90 mmol) and DMAP (20.4 mg, 0.167 mmol). The reaction mixture was allowed to stir at ambient temperature for 24 h. Additional succinic anhydride (45.6 mg, 0.455 mmol) was added and the reaction mixture was stirred for 7 h. The resultant solution was diluted with 2 N HCl (15 mL) and water (10 mL) and allowed to stir for 10 min. The resultant suspension was filtered and the collected solid was dried to give the title product (460 mg, 71%); MS *m/z*: 428 (M+H)^+. 1^H NMR (400 MHz, DMSO-*d*_6_) δ 12.16 (s, 1H), 10.14 (s, 1H), 8.47 (s, 1H), 8.17 (d, *J* = 0.7 Hz, 1H), 8.04 (d, *J* = 0.5 Hz, 1H), 7.88 (d, *J* = 0.7 Hz, 1H), 4.35 (s, 3H), 3.07 (q, *J* = 7.3 Hz, 1H), 2.73–2.58 (m, 1H), 2.47–2.40 (m, 2H), 2.05–1.05 (m, 12H).

#### α-cyclodextrin conjugate 16

A vial was charged with 4-(2-(4-((6-cyclohexyl-[1,2,4]triazolo[1,5-*a*]pyrazin-8-yl)amino)-1*H*-pyrazol-1-yl)ethoxy)-4-oxobutanoic acid (180 mg, 0.421 mmol), EDC (97 mg, 0.51 mmol), α-cyclodextrin (819 mg, 0.842 mmol) and DMAP (10.3 mg, 0.084 mmol). DMF (4.2 mL) was added and the reaction mixture was stirred at ambient temperature overnight. The reaction mixture was diluted with acetone (5 mL); a white precipitate formed which was collected via filtration. The crude material was purified by HPLC-MS to afford the α-cyclodextrin conjugate **16** as a white solid (130 mg, 22%). MS *m/z*: 1382 (M+H)^+^. ^1^H NMR (400 MHz, DMSO-*d*_6_) δ 10.13 (s, 1H), 8.47 (s, 1H), 8.15 (s, 1H), 8.04 (s, 1H), 7.90 (s, 1H), 5.69–5.33 (m, 12H), 4.78 (s, 6H), 4.59–4.16 (m, 11H), 4.08–3.95 (m, 1H), 3.91–3.82 (m, 1H), 3.82–3.71 (m, 6H), 3.71–3.50 (m, 16H), 3.47–3.30 (m, 10H), 2.70–2.51 (m, 5H), δ 1.92 (d, *J* = 11.9 Hz, 2H), 1.82 (d, *J* = 12.5 Hz, 2H), 1.73 (d, *J* = 11.5 Hz, 1H), 1.66–1.50 (m, 2H), 1.38 (d, *J* = 12.3 Hz, 2H), 1.32–1.18 (m, 1H).

#### β-cyclodextrin conjugate 17a

A vial was charged with 4-(2-(4-((6-cyclohexyl-[1,2,4]triazolo[1,5-*a*]pyrazin-8-yl)amino)-1*H*-pyrazol-1-yl)ethoxy)-4-oxobutanoic acid (211 mg, 0.494 mmol), β-cyclodextrin (1.12 g, 0.987 mmol), DMAP (12.1 mg, 0.099 mmol) and EDC (114 mg, 0.592 mmol). DMF (4.9 mL) was added. The reaction mixture was stirred at ambient temperature overnight. To the mixture was added acetone (10 mL). A white precipitate formed which was collected via filtration. Purification via prep-HPLC provided the title compound (40 mg, 5.2%); MS *m/z*: 1545 (M+H)^+^; ^1^H NMR (400 MHz, DMSO-*d*_6_) δ 10.13 (s, 1H), 8.47 (s, 1H), 8.15 (s, 1H), 8.03 (s, 1H), 7.90 (s, 1H), 5.85–5.67 (m, 14H), 4.81 (s, 7H), 4.40 (br s, 7H), 4.35 (s, 4 H) 4.31–4.20 (m, 2H), 4.17–4.09 (m, 1 H), 3.85–3.78 (m, 1H), 3.71–3.50 (m, 25H), 3.40–3.30 (m, 12H), 2.66–2.48 (m, 5H), 1.92 (d, *J* = 12.4 Hz, 2H), 1.82 (d, *J* = 12.8 Hz, 2H), 1.73 (d, *J* = 12.5 Hz, 1H), 1.57 (q, *J* = 12.6, 11.2 Hz, 2H), 1.38 (d, *J* = 12.7 Hz, 2H), 1.33–1.19 (m, 1H).

#### γ-cyclodextrin conjugates 18 and 19

4-(2-(4-((6-Cyclohexyl-[1,2,4]triazolo[1,5-*a*]pyrazin-8-yl)amino)-1*H*-pyrazol-1-yl)ethoxy)-4-oxobutanoic acid (460 mg, 1.08 mmol), EDC (289 mg, 1.51 mmol), γ-cyclodextrin (2.79 g, 2.152 mmol) and DMAP (26.3 mg, 0.215 mmol) were combined. DMF (3.0 mL) was added and the reaction mixture was stirred at ambient temperature overnight. Acetone (20 mL) was added to the reaction mixture and the resulting suspension was filtered and rinsed with additional acetone (2 x 10 mL). The collected solid was purified via HPLC-MS to yield two products as white solids, 2-*O*-γ-cyclodextrin conjugate **18** (258 mg, 14%); MS *m/z*: 1707 (M+H)^+ 1^H NMR (400 MHz, DMSO-*d*_6_) δ 10.14 (s, 1H), 8.47 (s, 1H), 8.15 (s, 1H), 8.04 (s, 1H), 7.92 (s, 1H), 6.06–5.36 (m, 16H), 5.13 (d, *J* = 3.8 Hz, 1H), 4.95–4.78 (m, 7H), 4.74–4.39 (m, 8H), 4.36 (s, 4H), 3.84–3.75 (m, 1H), 3.70–3.43 (m, 30H), 3.44–3.11 (m, 16H), 2.74–2.51 (m, 5H), 1.92 (d, *J* = 11.9 Hz, 2H), 1.82 (d, *J* = 12.6 Hz, 2H), 1.73 (d, *J* = 12.1 Hz, 1H), 1.65–1.51 (m, 2H), 1.39 (q, *J* = 12.5 Hz, 2H), 1.32–1.20 (m, 1H). 6-*O*-γ-cyclodextrin conjugate **19** (315 mg, 17%); MS *m/z*: 1707 (M+H)^+^. ^1^H NMR (400 MHz, DMSO-*d*_6_) δ 10.13 (s, 1H), 8.47 (s, 1H), 8.15 (s, 1H), 8.03 (s, 1H), 7.90 (s, 1H), 5.75 (br s, 16H), 4.89–4.84 (m, 8H), 4.46 (br s, 8H), 4.35 (s, 4H), 4.31–4.16 (m, 2H), 4.15–4.07 (m, 1H), 3.84–3.72 (m, 1H), 3.70–3.44 (m, 29H), 3.41–3.30 (m, 14H), 2.63–2.50 (m, 5H), 1.92 (d, *J* = 12.0 Hz, 2H), 1.82 (d, *J* = 12.8 Hz, 2H), 1.73 (d, *J* = 12.4 Hz, 1H), 1.68–1.50 (m, 2H), 1.38 (q, *J* = 12.6 Hz, 2H), 1.32–1.22 (m, 1H).

#### 4-((4-(4-((6-Cyclohexyl-[1,2,4]triazolo[1,5-*a*]pyrazin-8-yl)amino)-1*H*-pyrazol-1-yl)-2-methylbutan-2-yl)oxy)-4-oxobutanoic acid (40)

4-(4-((6-Cyclohexyl-[1,2,4]triazolo[1,5-*a*]pyrazin-8-yl)amino)-1*H*-pyrazol-1-yl)-2-methylbutan-2-ol (0.731 g, 1.98 mmol) and toluene (12 mL) were combined. Succinic anhydride (0.990 g, 9.89 mmol), 1-hydroxypyrrolidine-2,5-dione (0.455 g, 3.96 mmol), triethylamine (0.552 mL, 3.96 mmol) and DMAP (0.073 g, 0.59 mmol) were added. The suspension was heated to 120°C for 3 days in a sealed tube. The reaction mixture was cooled to room temperature and then DCM (20 ml) water (10 mL) and 1N HCl (10 mL) were added. The organic layer was separated and the aqueous portion was extracted with additional DCM (2 x 20 mL). The combined organic layers were dried over MgSO_4_ and concentrated. Purification by flash column chromatography (0–10% MeOH/DCM) provided the title compound (0.500 g, 54%); MS m/z: 470 (M+H)^+^. ^1^H NMR (400 MHz, DMSO-*d*_6_) δ 12.15 (s, 1H), 10.14 (s, 1H), 8.47 (s, 1H), 8.16 (s, 1H), 8.04 (s, 1H), 7.81 (s, 1H), 4.20–4.13 (m, 2H), 2.66–2.56 (m, 1H), 2.41 (s, 4H), 2.31–2.22 (m, 2H), 1.94–1.87 (m, 2H), 1.86–1.77 (m, 2H), 1.75–1.69 (m, 1H), 1.65–1.52 (m, 2H), 1.39 (s, 6H), 1.38–1.20 (m, 2H).

#### α-cyclodextrin conjugate 20

4-((4-(4-((6-Cyclohexyl-[1,2,4]triazolo[1,5-*a*]pyrazin-8-yl)amino)-1*H*-pyrazol-1-yl)-2-methylbutan-2-yl)oxy)-4-oxobutanoic acid (0.200 g, 0.426 mmol), α-cyclodextrin (0.829 g, 0.852 mmol) and *N*,*N-*dimethylpyridin-4-amine (10.4 mg, 0.085 mmol) were combined with DMF (4.0 mL). EDC (0.122 g, 0.639 mmol) was added and the reaction mixture was stirred at ambient temperature for 17 h. Acetone (5 mL) was added and the resultant precipitate was filtered. The collected solid was purified by HPLC-MS to provide the α-cyclodextrin conjugate **20** (0.140 g, 23%); MS m/z: 1425 (M+H)^+^. ^1^H NMR (400 MHz, DMSO-*d*_6_) δ 10.11 (s, 1H), 8.47 (s, 1H), 8.18 (s, 1H), 8.15 (s, 1H), 8.04 (s, 1H), 7.83 (s, 1H), 5.60–5.34 (m, 12H), 4.84–4.74 (m, 7H), 4.45 (br s, 5H), 4.34–4.27 (m, 1H), 4.25 (s, 1H), 4.21–4.10 (m, 2H), 3.89–3.82 (m, 1H), 3.81–3.72 (m, 7H), 3.71–3.52 (m, 16H), 3.47–3.30 (m, 10H), 2.69–2.57 (m, 1H), 2.55–2.49 (d, *J* = 6.7 Hz, 3H), 2.29–2.17 (m, 2H) δ 1.91 (d, *J* = 10.9 Hz, 2H), 1.82 (d, *J* = 12.8 Hz, 2H), 1.73 (d, *J* = 12.2 Hz, 1H), 1.59 (q, *J* = 12.1, 10.9 Hz, 2H), 1.46–1.33 (m, 8H), 1.32–1.18 (m, 1H).

### Biology methods

#### Cellular assays: Mouse macrophage differentiation assay

Mouse bone marrow derived macrophages (BMDM) were isolated as previously described[53] from C57BL/6 female mice. The isolated cells were washed using standard growth medium (RPMI (Gibco cat# 11875–093) with 2 mM L-glutamine (Gibco cat# 25033), 1% Pen/Strep (Gibco cat# 15140), 10% heat inactivated fetal bovine serum (HI-FBS, Gibco cat# 10438), and 0.1% beta-mercaptoethanol (BME, Gibco cat# 21985–023)), and filtered through a 100 μM cell strainer (Falcon cat# 352360). Cells were counted, re-suspended in growth medium, and plated at 1x10^5^ viable cells per well in a 96 well plate (Corning cat# 3603). Inhibitors were added at various concentrations to the cells, and incubated at 37°C/5% CO_2_ for 30 minutes. Recombinant mouse macrophage colony stimulating factor (M-CSF, R&D cat# 416-ML-010) was added to the plates at a final concentration of 50 ng/μL. Plates were transferred to a 37°C/5% CO_2_ incubator for 5 days. On the fifth day, supernatant was removed from each well and Cell Titer Glo (Promega cat#G7571) was added. Plates were covered from the light and incubated at room temperature for 10 minutes before reading the luminescence on an EnVision plate reader (PerkinElmer).

#### Broad kinase profiling method

TR-FRET-based binding displacement studies using active site probes were used to assess kinome selectivity of inhibitor molecules.[54] The concentrations of components for each purified kinase were based on optimized conditions with ranges as follows: kinases (1.25–12.5 nM), Oregon Green-labeled fluorescent probes (2x K_D_ of probe to the particular kinase; range of 1–200 nM; in-house), terbium anti-His or anti-GST antibody (0.5–2 nM). The reaction buffer was diluted to 1x using ddH_2_O and contained 1 mM DTT.

Compounds were typically tested from 0.0001 to 10 μM in 10x dilutions. Reactions were carried out in a 20 μL volume in 384-well plates by combining kinase, probe, antibody, and test compound and allowing the reaction to come to equilibrium (incubation of 2 h). TR-FRET was then measured on a PerkinElmer Envision plate reader (excitation at 340 nm; emission at 520 and 495 nm). Competition by the test compound versus the fluorescent probe for the ATP binding site of the kinase was quantified by calculating the IC_50_ value based on a four-parameter logistic curve fit.

#### Kinases measured in the in-house panel of kinases with compounds 1 and 4–6

ACVR1, ALK, Abl, Akt1, Aurora1, Aurora2, BRAF, BTK, CAMK1D, CAMK2A, CAMKK2, CDK2, CDK7, CDK8, CDK9, CDK11, CLK2, CSF1R, Ck1 alpha1, cMET, DDR1, DYRK1B, DYRK1A, EGFR, Erk2, FAK, FGFR1, Flt1, Fyn, GRK5, Gsk3a, Gsk3b, IGF1R, IKKE, InsR, JAK2, JAK3, JNK1, JNK2, Kdr, LTK, Lck, MAP2K3, MAP3K10, MAP4K1, MAP4K2, MAP4K4, MEK1, MEK2, MST1, Nek2, p38 alpha, PAK4KD, PDGFRA V561D, PDGFRB, PKA, PKCtheta, PKCzeta, PKG1A, Pim1, Pim2, Plk3, Prkcn, RET, RIPK2, Rock1, Rock2, Rsk2, SGK1, SIK1, STK16, STK33, Src, Syk, TAOK2, TBK1, TNK2, TYRO3, TrkA, TrkB, TrkC, Wee1, ZipK.

#### Per rectal (PR) dosing

Prior to dosing, mice were anaesthetized with Isoflurane and fecal material was removed from colons by irrigating with 0.5 mL of PBS using a syringe fitted with a 22 gauge flexible gavage needle. After a 20 min wake up period, mice were again anesthetized and drug was administered in a volume of 0.1–0.2 mL using a syringe fitted with a 22 gauge flexible gavage needle. Mice were then placed in the anesthesia chamber for 40 min in a supine position with hind end elevated to ensure drug remained in colon. After 40 min, mice were removed from the chamber and blood sampling over time (via tail nick) began.

#### Pharmacokinetic studies of active parent in mice

Male CD1 mice on Teklad Global Diet 2014 (Envigo Teklad, Madison, WI) (n = 2–3 per study) received a single IV, oral or intraperitoneal (IP) administration of compounds 1, 4, 5 or 6 at 1 mg/kg in 2:5:20:73 (v/v) DMSO:Tween 80:PEG-400:D5W dosed at 10 ml/kg. Serial blood samples were taken over 24h and frozen at −20°C until analysis. Pharmacokinetic parameters after IP or PO dosing were calculated by noncompartmental methods, while pharmacokinetic parameters after IV dosing were calculated by compartmental methods using WinNonlin (version 5.2, Certara USA Inc., St. Louis, MO). Mean parameters were determined from 1–3 separate studies. FaFg was determined as F/F_h_ (compound **6**) or as the ratio of AUC after an oral dose to that after an IP dose (AUC_oral_/AUC_ip_).

#### Oral administration of active parent or prodrugs to mice

Active Parent–Male CD1 mice (N = 3) received a single oral administration of compound 1 at 30 mg/kg prepared as a nanosuspension in 2% HPC-SL, 0.2% SDS in water and dosed at 10 ml/kg. Active Parent Prodrugs–Male CD1 mice or female C57BL/6 mice (N = 3) received a single oral adminstration of prodrug at 30 mg/kg active parent equivalent in either 10% Cremophor EL + 90% 1% Na-carboxymethylcellulose (NaCMC) at 10 ml/kg or as a nanosuspension in 2% HPC-SL, 0.2% SDS in water at 10 ml/kg.Blood samples were taken over 11 hours, and colon samples were taken at 3, 6 and/or 11 hours post dose. Mice received either Teklad Global Diet 2014 (Envigo Teklad, Madison, WI or LabDiet #5010 (LabDiet, St. Louis, MO).

#### Colon tissue preparation for determination of drug exposure

Due to the inherent risk of fecal or PR-dosed material contaminating true levels of colon tissue exposure, careful flushing of colon tissue for drug analysis was required. Upon necropsy, a section of mouse colon was removed, and flushed with PBS until no visible fecal material was observed. Subsequently, a 1–2 cm section of colon was blotted dry, weighed and snap-frozen in liquid nitrogen.

#### Homogenization of colon tissue

Colon tissue (~50 mg) was diluted 10x in Veterinary Sterile Water for Irrigation (USP by Abbott), then 1.4 mm zirconium oxide beads were added and tissue was homogenized using an Omni Bead Ruptor 24 (Omni International, Kennesaw, GA). Aliquots of colon homogenate samples and a set of colon standards were pipetted in 96-well plates and subjected to protein precipitation extraction (with acetonitrile + internal standard) on a Microlab Star robot (Hamilton Robotics, Reno, NV). Plates were then mixed on a MixMate (Eppendorf, Hamburg, Germany) for 1 min at 1600 rpm and centrifuging for 5 min at 4000 rpm in 5810 R Centrifuge (Eppendorf, Hamburg, Germany). Supernatant was transferred into new plates and diluted with LC-MS/MS mobile phase.

#### Quantification of drug

Spiked standards were prepared in the appropriate matrices. All unknown samples and spiked standards were combined with acetonitrile containing internal standard, vortexed, and centrifuged to pellet the proteins. Supernatants were used for subsequent analysis by LC-MS/MS. The mobile phase consisted of acetonitrile with 0.1% formic acid, and water with 0.1% formic acid at a flow rate of 0.8 mL/min. Analytes were separated using reverse phase chromatography with a fast gradient on a 30 x 2.1 mm Fortis C18 Pace 5 mm column, prior to analysis on a Sciex API5500™ mass spectrometer. Peak areas were determined using Sciex Analyst™ 1.6 software. Actual concentrations were calculated by regression analysis of the peak area ratio (parent / internal standard) of the spiked standards versus concentration.

#### Chronic dosing study in healthy mice

On day 0, healthy C57BL/6 female mice (Taconic) were dosed BID with compound **2** and continued dosing until Day 7. Compound **2** was administered in a formulation of 10:90 (w/w) Cremophor EL: 1% sodium carboxymethyl cellulose. On day 1, ~12 h after a single dose, a cohort of healthy mice was sacrificed for blood, liver, and colon exposure. On Day 7, 12 h post last dose, the remaining cohort was sacrificed for blood, liver, and colon exposure, representing 7 d BID dosing.

#### Mouse DSS model of colitis

On day 0, super prophylactic dosing of compound **2** began on C57BL/6 female mice. On day 7 DSS was administered via 3% DSS (Dextran Sulfate Sodium, MP Biomedicals cat# 160110) in drinking water. At this time compound **3** dosing initiated. On day 14, animals were sacrificed for PK and efficacy measurements (~11 h post-dose for compound **2**, 3 h post-dose for compound **3**).

### Statistical analysis

Statistical analyses of *in-vivo* pharmacodynamic and efficacy data were performed as follows. D'Agostino & Pearson normality test was first used to test data for Gaussian distribution. If all groups passed normality test, a One-way ANOVA was used with Dunnett’s multiple comparisons test to compare treatment groups to Vehicle group. If not all groups passed normality test, a Kruskal-Wallis nonparametric ANOVA was used, with Dunn’s multiple comparison test to compare treatment groups to Vehicle group. Levels of significance compared to Vehicle are annotated as follows: * = p<0.05, ** = p<0.01, *** = p<0.001, **** = p < 0.0001. Optimal number of mice per group was determined using power analysis; N = 10 mice per group at 80% power predicts statistically significant detection of ≥ ~40% reduction in colon IBA1+ and ≥ ~75% reduction in colon erosion length comparing treatment groups to Vehicle group. Naïve groups were omitted from statistical analyses.

### Ethics statement

Animal studies were conducted under a program accredited by the Association for Assessment and Accreditation of Laboratory Animal Care. All animal studies were reviewed and approved by AbbVie Bioresearch Center’s Institutional Animal Care and Use Committee.

## Abbreviations

ALT: alanine aminotransferase
AST: aspartate aminotransferase
BME: Beta-mercaptoethanol
BMDM: bone marrow derived macrophages
CK: creatine kinase
CD: Crohn’s disease
CSF1R: colony stimulating factor 1 receptor
GI: gastro-intestinal
HI-FBS: heat inactivated fetal bovine serum
IBD: inflammatory bowel disease
M-CSF: macrophage colony stimulating factor
MW: molecular weight
PO: per oral
PR: per rectal
RA: rheumatoid arthritis
tPSA: total polar surface area
